# Human robotic surgery with intraoperative tissue identification using rapid evaporation ionisation mass spectrometry

**DOI:** 10.1038/s41598-023-50942-3

**Published:** 2024-01-10

**Authors:** Eftychios Manoli, James Higginson, Neil Tolley, Ara Darzi, James Kinross, Burak Temelkuran, Zoltan Takats

**Affiliations:** 1 Department of Metabolism, Digestion and Reproduction, Imperial College London, London, UK; 2 Department of Surgery and Cancer, Imperial College London, London, UK; 3 The Hamlyn Centre for Robotic Surgery, Imperial College London, London, UK; 4 Laboratoire Protéomique, Réponse Inflammatoire et Spectrométrie de Masse (PRISM), Univ. Lille, INSERM U1192, Lille, France

**Keywords:** Mass spectrometry, Medical and clinical diagnostics, Translational research

## Abstract

Instantaneous, continuous, and reliable information on the molecular biology of surgical target tissue could significantly contribute to the precision, safety, and speed of the intervention. In this work, we introduced a methodology for chemical tissue identification in robotic surgery using rapid evaporative ionisation mass spectrometry. We developed a surgical aerosol evacuation system that is compatible with a robotic platform enabling consistent intraoperative sample collection and assessed the feasibility of this platform during head and neck surgical cases, using two different surgical energy devices. Our data showed specific, characteristic lipid profiles associated with the tissue type including various ceramides, glycerophospholipids, and glycerolipids, as well as different ion formation mechanisms based on the energy device used. This platform allows continuous and accurate intraoperative mass spectrometry-based identification of ablated/resected tissue and in combination with robotic registration of images, time, and anatomical positions can improve the current robot-assisted surgical platforms and guide surgical strategy.

## Introduction

Technological progress is continuously improving the precision of surgery, enabling more accurate and complete removal of pathological tissue, whilst preserving adjacent critical structures and healthy, functional tissue^1^. Surgical energy devices such as surgical lasers can control tissue removal down to micrometre resolution. The combination of such surgical tools with robotics offers increased accuracy of dissection through scaling of surgical input and elimination of physiological tremor. These advanced technologies enhance the precision of removal of the target tissue, but the decision-making process in selecting the target tissue to be removed currently relies on the surgical sensorium, the accuracy of which determines the actual precision limit of surgery.

Current surgical strategy in Ear, Nose, and Throat (ENT) / Head and Neck (H&N) subspecialty procedures rely on the use of pre-operative imaging and intraoperative visual and haptic feedback. Postoperative histological analysis guides the need for any subsequent intervention. In conventional open operations, surgeons identify and differentiate tissue by sight, and using haptic feedback, including the kinaesthetic sense of how tissues respond and deform in response to forces applied, and the tactile sense of how the tissues feel in contact with the surgeon’s hands^2^. These senses act together to play a key role in surgical decision-making, particularly when distinguishing pathology, tissue types, and surgical planes. This presents a key limitation of the state of the art in surgical robotics: the transition to robotic surgery offers an enhanced visual experience, but current robotic instruments and consoles are not capable of providing haptic feedback, highlighting the need for additional sensors. In some contexts, intraoperative decision-making can be assisted by sending histological specimens for laboratory analysis—known as ‘frozen section histology’. In addition to being user-dependent, costly, and time-consuming, this approach only gives local information for selected areas and therefore is mostly used for margin assessment where malignant tumours are being removed.

Intraoperative ambient Mass Spectrometry (MS) techniques have a potential role in augmenting the robotic surgical sensorium, by offering the surgeon continuous instantaneous information about the freshly resected tissue. These technologies, including the iKnife^3^, the SpiderMass^4^, the MassSpec Pen^5^ and the Picosecond InfraRed Laser (PIRL) MS^6^, generate samples in-situ for immediate MS analysis. The MassSpec Pen performs surface tissue extraction using a water droplet and sends the extract into the MS instrument using fluidics. The total analysis time (including the process of analyte extraction and transfer, MS analysis, and tissue recognition) is completed in 10 s, and the risk of sample carryover (especially in endoscopic or laparoscopic surgeries) is high^7^. Despite these limitations, this technology offers a non-invasive approach to intraoperative tissue diagnostics and was tested and validated in the human breast, thyroid lung, pancreatic and ovarian cancer tissue^5,8,9^.

In contrast, the iKnife and related technologies (SpiderMass and PIRL-MS) utilize the aerosol routinely generated during procedures as a by-product of surgical energy devices.

In the case of SpiderMass, a fibred IR laser through a micro-sampling probe is used, which causes resonant excitation of the endogenous water molecules which can then be aspirated and transferred to the mass spectrometer for analysis^10^. This technology has been applied in vivo on a volunteer human’s healthy skin^4^ and for the diagnosis of sarcomas in dogs^11^. In the case of the PIRL-MS, a handheld PIRL probe is used to ablate the tissue using a commercial solid state picosecond mid IR laser. The generated plume is transferred through a 2 m long Tygon tube, to the heated inlet capillary of a commercially DESI-MS source^12^. Tissue-specific lipid profiles were generated within 5–10 s after the ablation of tissue. This technique was validated in human breast cancer xenografts130 and medulloblastoma xenografts^6,13^, however, has not yet been applied for in vivo tissue identification. Although there have been demonstrations of these technologies coupled to a robot^14,15^, to our knowledge, none have been used in robotic surgery on a human patient.

For the iKnife technology, the generated aerosol is ionised by a rapid evaporative ionisation mass spectrometry (REIMS) source coupled to a mass spectrometer for continuous analysis. Unlike the aforementioned technologies, the REIMS system utilises the aerosol produced by the existing surgical resection tools without the need to introduce a new energy source or any other instrumentation to the surgical area for diagnosis, eliminating the need to switch instruments during surgery.

. Another advantage is the immediate feedback (< 1 s time delay) that is continuously provided during surgical resection, which helps the adaptation of this intraoperative tissue analysis to surgery. Through the MS analysis of cellular lipids which are tissue-specific and biologically significant, REIMS provides a unique biochemical insight to the surgeon in real-time, without interrupting the surgical technique or workflow or extending operative/anaesthetic time.

**Figure 1:**
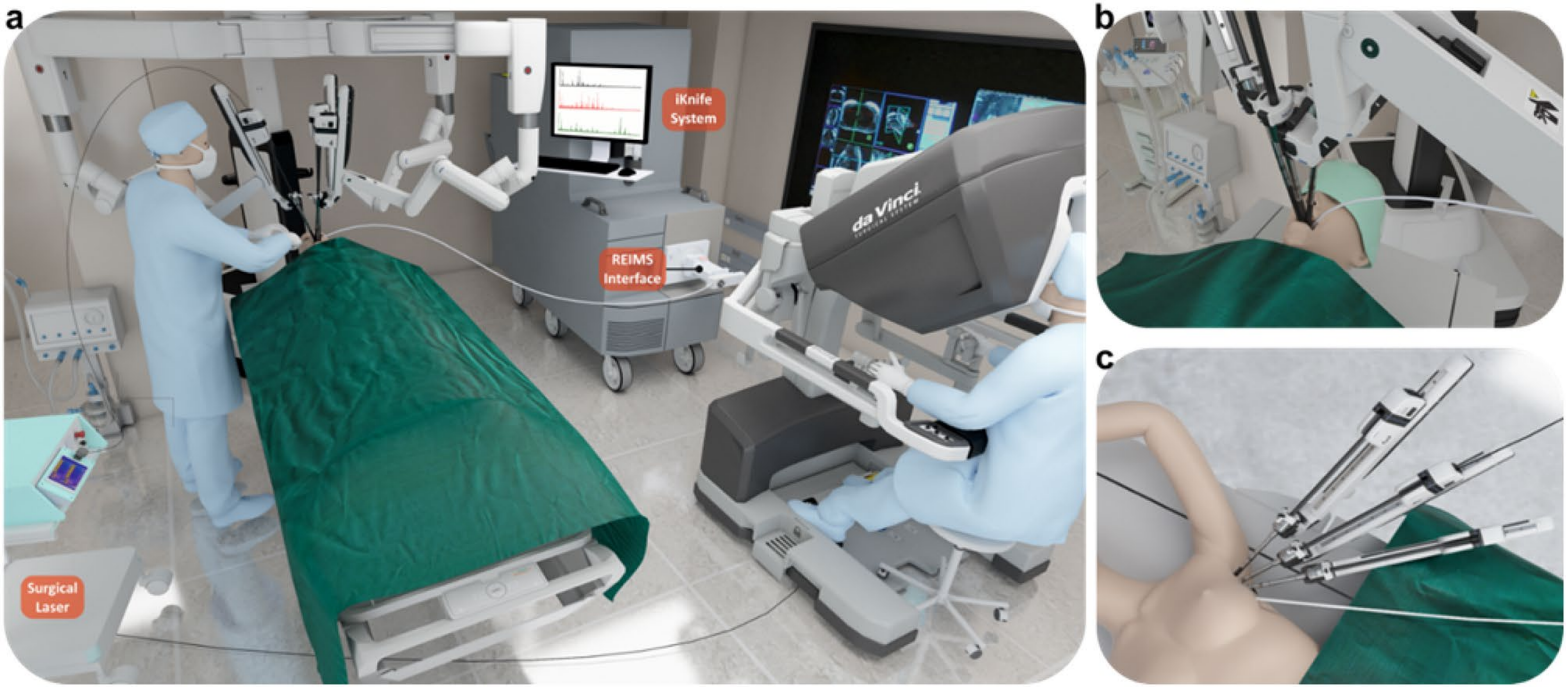
Illustration of the operating theatre set-up with iKnife/REIMS instrumentation and surgical robotic system. (**a**) Simplified intraoperative set-up of the first in human-robotic iKnife cases. The da Vinci Si robotic system (Intuitive Surgical, CA, USA) was used including the surgeon’s operating console. The iKnife system was equipped with a REIMS interface where analysis of surgical aerosol occurred. The surgical aerosol was collected from the working surgical environment and transferred to the iKnife instrument using a 3 mm internal diameter polytetrafluoroethylene (PTFE) aspiration tube. (**b**) Close-up illustration of the robotic arms and the iKnife aspiration tube during the TORS cases. For tissue ablation, a 2 µm thulium laser fibre (RevoLix, CA, US) was used. (**c**) Close-up illustration of the robotic arms and the iKnife aspiration tube during the transaxillary parathyroidectomy case. For tissue cutting and coagulation, the Harmonic Ace Curved Dissector (Ethicon Endo-surgery, part of Johnson and Johnson, OH, US) was used.

In this study, we demonstrate the first-in-human utilization of a MS tissue identification system (REIMS) with a robot, to enhance the surgical sensorium (Fig. 1a). We use a 2 µm wavelength thulium laser in two Transoral Robotic Surgery (TORS) sleep apnea cases (Fig. 1b), and the Harmonic Ace Curved Dissector in a transaxillary robotic-assisted parathyroidectomy case (Fig. 1c), demonstrating the formation of a largely aqueous aerosol that is chemically representative of the tissue. Consequently, this aerosol analysed by MS using the REIMS approach yields a tissue-aware MS-robotic guided surgical system.

## Results

### Surgical aerosol evacuation system

**Figure 2:**
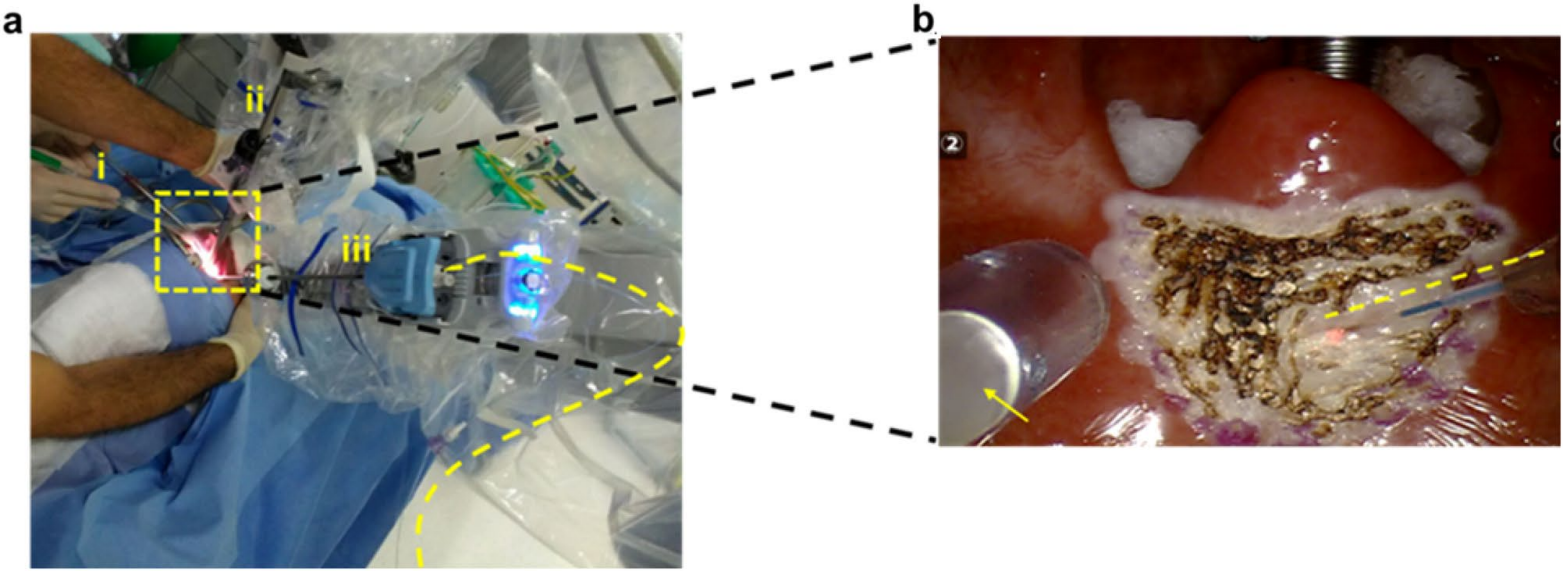
iKnife-TORS intraoperative set-up and close-up view of the surgical working environment. (**a**) (i) Patient-side surgical assistant providing external suctioning (ii) Central robotic arm holding a 12 mm 30° up stereoscopic endoscope (iii) Right 5 mm robotic arm holding a 2 µm thulium laser fibre (dashed curved yellow line). (**b**) Surgeon’s view of the soft palate ablation. The modified external suction tool (yellow arrow) facilitates aspiration of the smoke generated by the thulium laser (dashed yellow line) into the mass spectrometer.

Adequate intraoperative smoke evacuation is crucial, especially in enclosed surgical sites such as the oral cavity, where smoke may obscure the surgeon’s view. Efficient smoke evacuation must be kept on all the time during the resection/ablation, which helps continuous data collection and hence uninterrupted tissue identification with REIMS. In order to aspirate the smoke generated and direct it into the MS instrument, a 3 mm internal diameter PTFE tubing was attached on the external suction with sterile tape (Fig. 2a). As this external suction is also used to extract liquids, which if aspirated to the MS analyser would disable it, the REIMS suction tube was slightly recessed relative to the main suction (Fig. 2b). To further reduce the risk, the REIMS interface was also equipped with a liquid trapping system. When any liquid traveling along with the surgical aerosol is identified (through various sensors), the system automatically stops any MS function as a safety precaution. The suction was manually controlled by the assistant surgeon next to the patient’s head (Fig. 2a). The suction tool was positioned on the left side of the patient’s mouth allowing the surgeon to have a clear view of the tissue ablation area, working space, and surgical instruments. The suction tool had to be at an optimal distance from the ablation point allowing sufficient smoke to be transferred into the MS, as demonstrated in Movie S1. Both the external suction tool and the iKnife suction were turned on, causing the aerosol to split into two ways, with the aerosol predominantly going into the external suction tool, which is closest to the ablation point, while allowing consistent sample capture by the REIMS aspiration tube to generate continuous MS signal. Surgical aerosol was successfully captured from the left side of the mouth area (Fig. 2b). During surgery, the surgeon (NT) operated, as usual, using the laser, which he maneuvered by the robotic arm. The laser fibre was used in the “near-contact” method keeping it about a millimetre away from the tissue to achieve ablation of tissue with a simultaneous bleeding control (haemostasis) which is observed as bleaching of the tissue (Movie S1). The only noticeable difference for the surgeon, if any, was the partially visible presence of the tip of the opaque PTFE tube sitting on top of the transparent external suction tubing within the operative field (as seen in the bottom left corner of Fig. 2b).

### Laser-assisted (LA): REIMS in TORS

During TORS for sleep apnea, the surgeon ablated tissue from different anatomical subsites in the oropharynx using the surgical laser. The oropharynx is lined throughout with mucosal epithelium. At each subsite, including the soft palate, the base of the tongue, and epiglottis, the basic tissue histological type is the same: stratified squamous epithelium. However, the epithelium at each subsite has its own distinct histological characteristics. The soft palate is lined with thin, soft, non-keratinised stratified squamous epithelium (palatal NKSSE). The anterior surface of the epiglottis is also covered with thin, non-keratinized stratified squamous epithelium which is closely attached to the underlying fibroelastic cartilage (epiglottic NKSSE). In contrast, the squamous epithelium lining the posterior tongue is much thicker keratinized stratified squamous epithelium (lingual KSSE).

**Figure 3:**
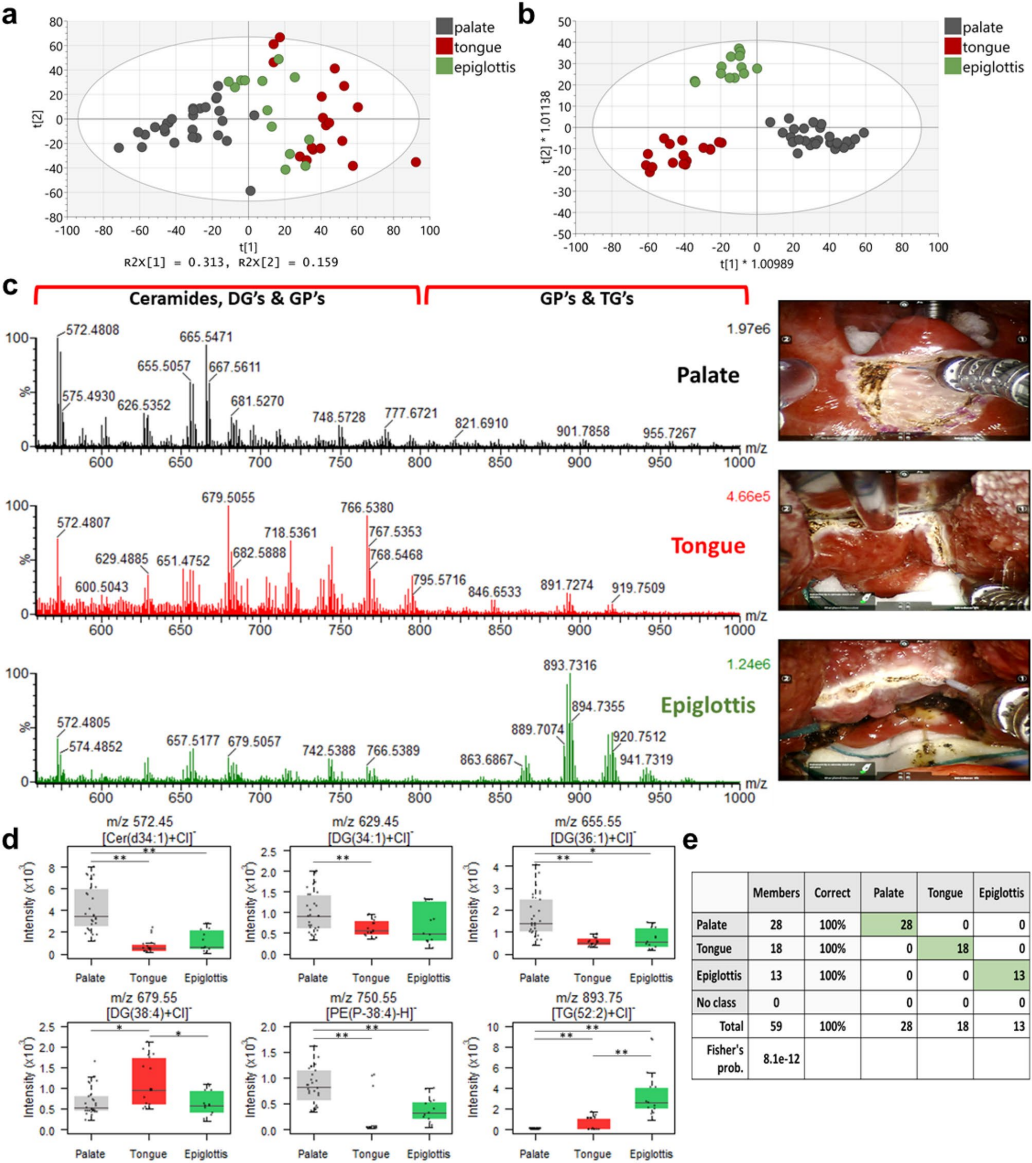
Statistical analysis and spectra interpretation of the iKnife-TORS data. (**a**) PCA plot of the palate (grey), tongue (red), and epiglottis (green) tissue using the thulium laser during the TORS cases within the *m/z* range of 560–1000. Each sampling point represents a unique area sampled for each tissue type, with each sampling point representing a scan resulting in a single spectra. Each sampling point/area was selected based on the relative intensity of ions observed in the *m/z* range of 560–1000. The most significant variation was shown by PC1 = 31.3% and PC2 = 15.9%. The tolerance ellipse of the two-dimensional score plot was based on Hotelling’s T2 with a significant level set to 0.05. (**b**) OPLS-DA plot of different tissues during the TORS cases showing a clear separation between different tissue types in the *m/z* range of 560–1000. For the model two predictive and one orthogonal component were used. (**c**) Averaged REIMS spectra (*m/z* range of 560–1000) and corresponding anatomical images during the ablation of palate, tongue, and epiglottis. (**d**) Box plots showing the intensity of some of the most statistically significant lipid metabolites responsible for separating palate, tongue, and epiglottis. The box represents the interquartile range with the median shown. The whiskers represent the range of data points. Raw data are represented using jitter points. ANOVA was performed between the groups where p values were calculated after correction using the FDR method (with *p < 0.001 and **p < 1e−5). (**e**) An accuracy of 100% was observed on a left-out test set when using an OPLS-DA model (with two predictive and one orthogonal components) to differentiate between the different tissue types of the palate (n = 28), tongue (n = 18), and epiglottis (n = 13) trained on all other spectra. All the observations were assigned to the nearest class, without using a threshold. (Cer-Ceramides, DG-Diglycerides, PE-Phosphatidylethanolamines, GP-Glycerophospholipids, TG-Triglycerides, *m/z*-mass-to-charge ratio, R^2^Y-The fraction of squares of all the y-variables, Q^2^- The fraction of the total variation of all the x and y variables).

The generated surgical aerosol was aspirated into the MS using the direct introduction method as described in Supplementary Information (Supplementary Text S1, Figs. S1–S2, Tables S1–S3). REIMS spectra were recorded in the *m/z* range of 100–1500 from the palatal NKSSE, the lingual KSSE, and NKSSE from the lingual surface of the epiglottis (Fig. S3), showing some distinct differences, especially in the *m/z* range of 560–1000 (where lipid metabolites are easily identifiable). PCA analysis (Fig. 3a) exhibited some marked clustering between sampling points from lingual KSSE (n = 18), palatal NKSSE (n = 28), and epiglottic NKSSE (n = 13) in the *m/z* range of 560–1000. The OPLS-DA model (Fig. 3b) showed a clear separation between the three tissue types based on the different metabolic profiles observed within the same *m/z* range, having a high predictive ability with R^2^Y = 0.89 and Q^2^ = 0.81.

The spectra of the 50 ions with the highest relative abundance in the *m/z* range of 560–1000 for all tissue types revealed some marked differences, showing features associated with abundant lipid species including glycerophospholipids, ceramides, and glycerolipids (Fig. 3c). Spectra similarities were also observed, with 15 ions (out of the 50 most intense ions) being present in all three tissue types (underlined in Tables S4–S6). During the analysis of the superficial mucous membrane in palatal NKSSE, 78% of the metabolites that putatively identified (including isotopes) were ceramides and diglycerides, especially observed in the *m/z* range of 550–850 (Table S4). The majority of these metabolites are ionized through the addition of a chloride ion, (a common ionization mechanism observed in REIMS analysis^16^), with deprotonation and demethylation mechanisms representing only 14% of the ions observed. During ablation of palatal NKSSE, only the superficial oral mucosa and intermediate layers were affected, with the deeper muscle layers (e.g. palatopharyngeus muscle) remaining intact. In addition, a very small number of glycerophospholipids (only 6% of the total putatively identified metabolites, Table S4) were observed.

A high number of glycerophospholipids, especially phosphatidylethanolamines and phosphatidylcholines, were observed in the lingual KSSE and epiglottic NKSSE spectra (the majority of them being with an ether linkage) in the *m/z* range of 700–800, accounting for 42% and 16% of the total metabolites observed respectively (Tables S5 and S6). For lingual KSSE and epiglottic NKSSE, a high-intensity triglyceride signal was observed in the *m/z* range of 850-1000 (Fig. 3c). In the case of epiglottic NKSSE, 20 features (including isotopes) were putatively identified as triglycerides, compared with lingual KSSE REIMS spectra where 8 ions were observed (Tables S5 and S6). The high triglyceride signal observed especially from the anterior surface of the epiglottic NKSSE (as well as the combination of the glycerophospholipid and triglyceride signal in tissue) is associated with the dissection of mucosal epithelium. This is in agreement with previous studies where a mixture of both mucosa and submucosa of the aerodigestive tract were being dissected^17^.

Univariate analysis revealed a variety of compounds including ceramides, diglycerides, phospholipids, and triglycerides, that were statistically significantly different on ANOVA between the palatal NKSSE, the lingual KSSE, and the epiglottic NKSSE (Fig. 3d). The ability of REIMS to distinguish the different histological tissue types during TORS cases using the distinctive lipid composition of each tissue was assessed, showing an accuracy of 100% on a left-out test set using an OPLS-DA model (Fig. 3e). The confusion matrix of separate test set (n = 19; palate = 8, tongue = 6 and epiglottis = 5) showing 100% accuracy can be found in Supplementary Information, Fig. S4) The validity of the OPLS-DA model was assessed using permutation randomisation tests (Supplementary Information, Fig. S5).

### REIMS in transaxillary parathyroidectomy

In addition to the non-invasive trans-oral approach, the surgical robot offers the possibility of minimal access surgery, in which access incisions can be made smaller and concealed in aesthetically inconspicuous areas. The transaxillary approach to the anterior neck is a prominent example of this, where an incision is made in the anterior axilla and subcutaneous dissection to the anterior neck creates a pocket that allows the robot to operate on the thyroid or parathyroid glands^18,19^. This approach is only possible with the use of the robot due to the very limited space and the risk of causing significant damage to the overlying skin^20^.

In this case, the patient was undergoing a transaxillary parathyroidectomy for a benign parathyroid adenoma of the left superior parathyroid gland. Due to the limited space of the transaxillary treatment approach, a different aerosol capture technique was developed, where the REIMS aspiration tube (i.d 3 mm, PTFE) was attached inside (and underneath) the special retractor which was mounted to the other side of the operating table (Fig. S6). For aspiration of the surgical aerosol, the direct introduction method was used as described in Supplementary Information (Supplementary Text S1, Figs. S1–S2, Tables S1–S3).

**Figure 4:**
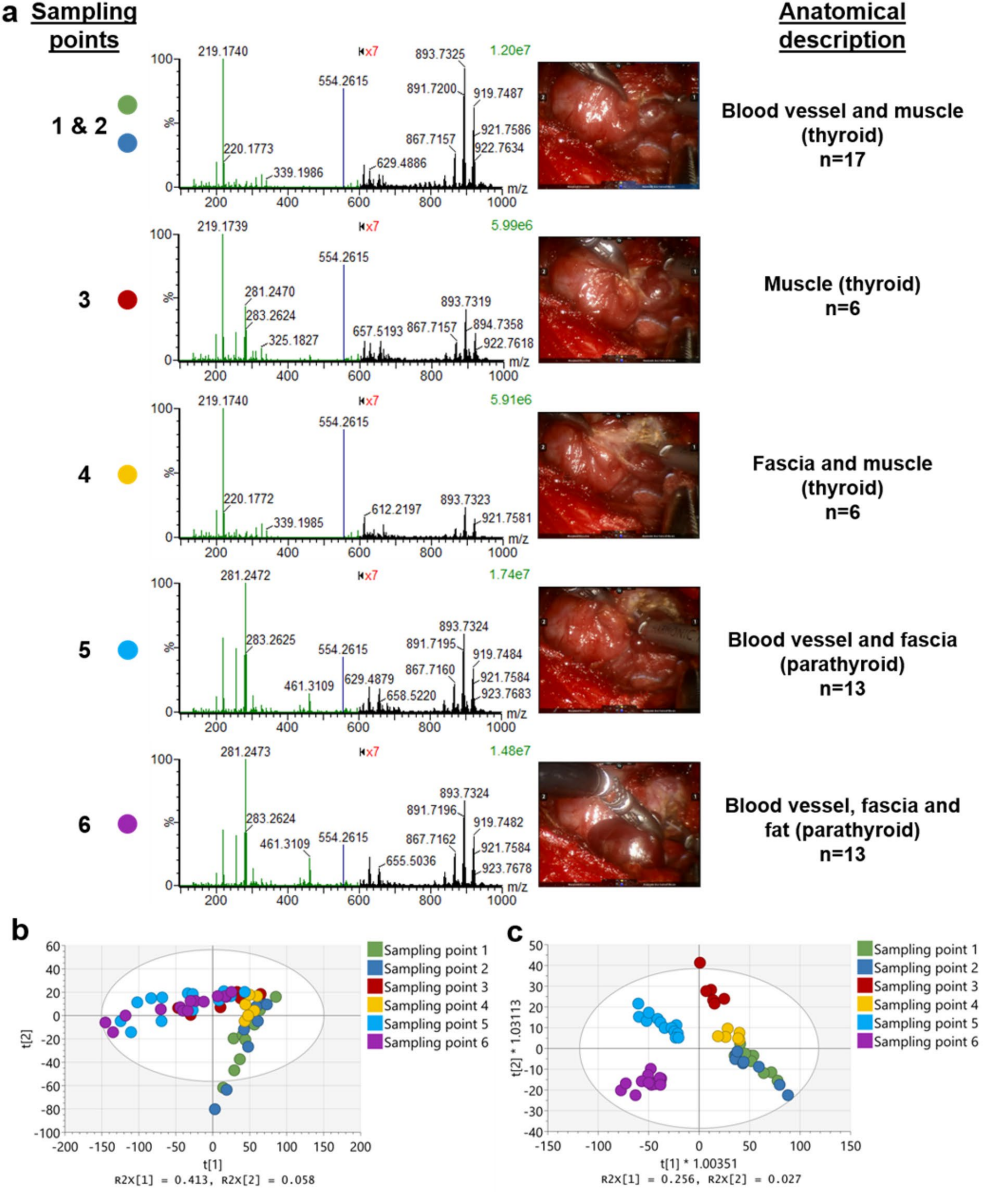
Statistical analysis and spectra interpretation of the iKnife-Parathyroidectomy data. (**a**) Averaged REIMS spectra with the corresponding tissue sampling/dissection points within the *m/z* range of 100–1000. Each scan corresponds to a generated spectra (n). Visual tissue interpretation was performed for the different sampling points. Each colour dot represents a different sampling point also represented in Fig. 4b, c. Sampling was done using the Harmonic Ace Curved Dissector (Ethicon, JnJ, US) attached to the right robotic arm. For better visualization, the *m/z* range of 600–1000 was magnified (×7), showing spectra differences for each tissue type during the dissection process. (**b**) PCA (with PC1 = 41.3% and PC2 = 5.8%) and c) OPLS-DA plots of the different sampling points of the iKnife-parathyroidectomy case, in the *m/z* range of 100–1000. The tolerance ellipse of the two-dimensional score plots was based on Hotelling’s T2 with a significant level set to 0.05. For all the bins centering and auto-scaling to Unit Variance (UV) was used. Each dot represents spectra for each of the coloured sampling points. For the OPLS-DA plot, two predictive and one orthogonal component were used. There is a clear separation between the different sampling points, with the sampling points 1&2 clustering together, suggesting lipid similarities associated with the dissected tissue.

During the procedure, six sampling points were made using the Harmonic device generating 55 spectra in total (Fig. 4a). At first, surrounding tissue was dissected, and the parathyroid gland was exposed followed by the complete removal of the gland as presented in the Movie S2. For each of the sampling points, a visual tissue anatomical interpretation was made with the corresponding spectra shown in Fig. 4a. Different tissue types were identified including blood vessels, muscle, and fascia as part of the dissection of thyroid tissue (sampling points 1–4) and blood vessels, fascia, and fat being part of the parathyroid tissue (sampling points 5 and 6). PCA plot showed some overlap clustering between the different sampling points (Fig. 4b), whereas the OPLS-DA plot showed a clear separation of the sampling points based on the different metabolic profiles observed within the *m/z* range of 100–1000 (Fig. 4c). The model distinguishes the majority of the sampling points (except from 1 and 2 suggesting spectra similarities), showing high inter-group compared to intra-group variance. As a result, the model had a moderate predictive ability with R^2^Y = 0.38 and Q^2^ = 0.26.

The spectra observed within the *m/z* range of 100–1000, showed features associated with abundant lipid species including mostly fatty acids, ceramides, and glycerolipids (Fig. 4a). It was found that there is a very low abundance of fatty acids in sampling points 1, 2, and 4 compared to the sampling points 3, 5, and 6 (Fig. S7), without that been associated with the number of spectra or the tissue type analysed (i.e. muscle part of the thyroid). The most statistically significant fatty acids, when comparing differences in relative abundance between the sampling points, include palmitic acid at *m/z* 255.2331 (*p* = 3.62e−07), linoleic acid at *m/z* 279.2334 (*p* = 3.77e−07), oleic acid at *m/z* 281.2373 (*p* = 2.64e−07), stearic acid at *m/z* 283.2625 (*p* = 9.30e−08) and arachidonic acid at *m/z* 303.2335 (*p* = 2.61e−07) (Fig. S7). In addition, a contaminant ion at *m/z* 219.15 was present in all the spectra (Fig. 4a) and is yet to be identified. When exploring the relationship between the *m/z* 219.15 with each of the fatty acids above, it was found that all combinations were negatively correlated (Fig. S8), with the high abundant contaminant affecting the ionization efficiency causing signal suppression to the low mass metabolites next to it. As a result, when the signal intensity of the ion at *m/z* 219.15 increases, the intensity of fatty acids decreases, and vice versa.

In the *m/z* range of 600–1000, the spectra from the different sampling points present a similar biological signal (Fig. 4a), with most of the 40 highest intensity ions showing features associated with glycerolipids (Table S9). The majority of them (65%) were triglycerides (including isotopes) followed by 18% identified as diglycerides, with all of the metabolites being ionised with the addition of chloride adduct. 15% of the ions were background noise peaks.

Following an S-plot analysis between the tissue structures associated with the thyroid and parathyroid in the *m/z* range of 600–1000, it was found that the putatively identified ions at *m/z* 722.5109 [PE(P-36:4)-H]- and at *m/z* 875.7710 [CE(32:4) + Cl]− contributing most to the group separation (Fig. S9a). The ion at *m/z* 722.5109 was found to be higher in abundance in the parathyroid tissue (sampling points 5 and 6), compared to the thyroid tissue (sampling points 1–4, Fig. S9b). The ion at *m/z* 875.7710 was found to be lower in abundance in sampling points 1, 2, and 4 and higher in abundance in sampling points 3, 5, and 6 (Fig. S9c), following the same intensity patterns with the fatty acids, previously observed. Triglyceride ions such as *m/z* at 891.7197 [TG(52:3) + Cl]−, *m/z* at 893.7324 [TG(52:2) + Cl]− and *m/z* at 919.7485 [TG(54:3) + Cl]− were found higher in abundance in sampling points 1 and 2 (blood vessels and muscle tissue) (Fig. S10), and differences in the relative abundance of triglycerides explain the high intra-group variation observed in the OPLS-DA model between sampling points 5 and 6 (Fig. 4c), where blood vessels, fascia, and fat tissue are dissected. We observed higher variability specifically in triglyceride’s signal intensity (Fig. S10) compared to other metabolites (i.e. fatty acids, Fig. S7) within the same sampling event, therefore this is not related to the burn duration, tissue type, or the number of data points acquired. We have previously encountered similar high variations on sampling data using the Harmonic device in biological tissues^16^ and this phenomenon needs to be further investigated.

## Discussion

The introduction of intraoperative tissue identification into robotic surgery is an important step in precision medicine, presenting immediate benefits and potential advancements for robotic surgery.

There are significant efforts to introduce haptic feedback to robotics^21^, a key sensing mechanism assisting surgeons with tissue identification and surgical planning, that is lost in transitioning to robotic surgery. Here, we demonstrate a platform where continuous MS-based tissue identification enhances the robotic surgical approach with a higher ambition than simply imitating the lost haptic sense. In the TORS cases, the surgical system was able to correctly classify all tissue types, with an overall diagnostic accuracy of 100% (confirmed by visual assessment). With the accuracy achieved in this work, we demonstrate that a molecular-aware robotic system can help navigate through complex anatomical layers with a high resolution in all directions, as the resolution can be defined by the well-controlled spot size and the depth of penetration of the laser beam. During the parathyroidectomy case, our results confirm the ability of REIMS to differentiate various tissue layers from a specific anatomical region within the same dissection event, based on details such as the presence of fat and blood vessels that are not easily distinguishable during surgery. The system brings depth awareness to robotic surgery, helps navigation through complex layers of anatomy, which is usually not visible to the naked eye, especially when the use of an energy source distorts the way the tissue looks.

While we investigate the benefits of introducing REIMS into robotic surgery in this first-in-human study, we acknowledge the fact that further exploration is needed. There are significant efforts for the creation of large, robust, histologically validated ex vivo spectral databases, facilitating the use of the technology in robotic surgery. As the spectral database gets richer to cover the identification of molecules for tissue around critical structures, such as myelin sheath of nerves or outer layers of blood vessels, the system presents an unprecedented opportunity to improve surgical precision and safety. This is possible by introducing safety mechanisms such as early warning or emergency shutdown, that could simply be integrated into the robotic system to avoid inadvertent damage to these structures during surgical interventions with the associated morbidities.

Along with the previously reported ex vivo*,* high accuracy identification of cancer tissue from the healthy tissue^22^, the feasibility demonstrated here will pave the way to several additional surgical benefits including highly accurate knowledge of tissue phenotype complementary to what eye can identify, improved precision in cancer resections, and reduction in duration of surgery eliminating intraoperative histopathology^23,24^. The continuous collection of tissue information during ablation of a large area performed in this case is a good indication that this technique could also be developed to scan a tumour bed to detect remnants of malignant tissue after tumour removal, an approach that may help achieve complete disease removal, especially with the aid of the robotic system. The robotic system here can be used in an autonomous way to scan such areas and register the position of any suspicious tissue, augmenting the real surgical site view of the surgeon, and yet we are one step away from this molecular aware robotic system to autonomously locate and ablate the suspicious cells until there is no signal from disease tissue. When combined with the information from the pre-operative scans, the recovery of the 3D structure of the surgical site, and the tracking of the biopsy sites using tools such as vision-based frameworks and surgical navigation systems^25,26^, the molecular aware robotic system is a major step forming the foundations of an increasing level of autonomy in robotic surgery.

One of the key factors that we envision will help the wider adoption of this technology by robotic surgeons is the fact that the integration does not require the addition of any specialised instruments to the surgical site. Adding and controlling a new tool in robotic surgery might be challenging, as it may require an additional robotic arm or replacement of an existing one to be used and maneuvered by the surgeon during an operation. In addition, the integration does not require any change in surgical technique or introduction of any new element obscuring the surgeon’s view. We showed that this approach is potentially applicable using various robot-compatible precision surgery energy sources having minimal thermal damage^27^, and for disparate anatomical locations. It is also worth mentioning that the use of laser energy, in addition to the benefits mentioned in the Supplementary Text S2 for this wavelength (2 µm), enables non-contact tissue ablation which is a significantly simpler motion to automate, especially when compared to other energy sources that need contact with tissue (which may also require another complex motion—cleaning of the tip) and complicated mechanical actuation motions such as grasping and retracting.

Predictions about advances in robotic surgery often focus on improvements in miniaturisation, integrated imaging, and haptics^28^. The use of robotic sensing or tissue recognition to deliver precision or targeted therapy has been anticipated only in vague terms^29,30^. However, the rapid growth towards minimally invasive precision surgery has opened the field in the development of advanced technological platforms using artificial intelligence and machine learning, aiming to combine computational and mechanical innovations, improving clinical decision making in an intraoperative setting^31,32^. Our capacity to digitally collect, interpret, and utilize visual information from advanced cameras enhances the capacity of the human eye in robotic surgery. The presented intraoperative sensing mechanism and the resulting tissue type awareness introduced to the robotic surgery is a crucial step moving from the current state of art robot-assisted surgery towards a more precise, faster, safer, and potentially more autonomous robotic surgery.

### Limitations

While this study was designed to show the feasibility of introducing REIMS into robotic surgery further studies are needed (with higher number of patients recruited) to validate these findings and assess the diagnostic accuracy of the iKnife technology in comparison with the current histopathology techniques. In the case where histopathological assessment is used (from a biopsy), the destructive nature of the energy device needs to be assessed and carefully designed experiments need to be made looking for example at depth tissue penetration and how is affected during the analysis using a specific energy device.

Another important aspect would be the impact of different parameters (during the in vivo data collection) on the biological signal/information and subsequently on the accuracy of tissue classification models. For example, the existence of saline contamination (f.e. on the mucosal surface) that is normally being used as an irrigation fluid (to clear items such as tissue debris and blood) as well as how the REIMS signal is affected when necrotic tissue is analysed (where dissection/ablation energy is applied repeatedly)). In addition, REIMS is currently adapted and used on an MS platform which is built for research purposes and allows to collect high-resolution data, therefore it is bulky and expensive. We anticipate the MS that will be used in theatres in the future, will not require high resolution (compared to the one used in this work) and sensitivity, resulting in lower cost and size.

## Methods

### Patients

Patients undergoing ENT/H&N robotic-assisted surgery were recruited at St Mary's hospital, UK as part of a prospective, observational cohort study. That includes two patients diagnosed with obstructive sleep apnea undergoing robotic-assisted uvulopalatopharyngoplasty and one patient undergoing a robotic left superior parathyroidectomy because of hypercalcemia. Ethical approval was obtained from NHS Health Research Authority (NRES Committee East of England—Cambridge East) with REC reference: 14/EE/0024 (IRAS project ID:124,247). All patients have given informed and written consent and all experiments were performed in accordance with relevant guidelines and regulations. Data were collected in course of two TORS and one parathyroidectomy procedure (Table S8).

### Surgical procedure

#### TORS

The DaVinci Si (Intuitive Surgical, CA, United States) surgical robotic system including the surgeon’s operating console was used. The cart was docked at an angle of 30°–45° relative to the base of the surgical bed. The patient was placed in the supine position with a head ring and shoulder roll for an optimal cervical extension. Both patients had nasotracheal intubation to allow the robotic instruments the best possible access. Uvulopalatopharyngoplasty was performed using a 2 µm thulium laser (RevoLix, CA, US) for the ablation of the tongue, palate, and epiglottis.

#### Parathyroidectomy

The patient was placed as described above and an incision was made in the patient’s axilla for the robotic access. The patient’s left arm was positioned to the ‘extended salute’ position (where the back of the patient’s hand rests on their forehead). An ultrasonic Harmonic Ace Curved Dissector (Ethicon Endo-surgery, part of Johnson and Johnson, OH, US) was attached to the robotic arm and used for tissue coagulation and removal of the parathyroid gland.

### Instrumentation

The surgical aerosols produced were transferred through the 3 mm internal diameter PTFE tubing and aspirated into a Xevo G2-S QToF mass spectrometer equipped with a REIMS interface (Waters Corporation, UK). Vapours produced were co-aspirated with a solvent solution of Propan-2-ol^33^ (Sigma-Aldrich, UK) containing Leucine Enkephalin (Sigma-Aldrich, UK) (10 ng/mL) as a lock mass reference compound with a flow rate set to 0.15 mL/min. Data were acquired in negative ionization mode. For the TORS cases ablation of tissues was carried out using a 2 µm thulium laser (RevoLix, CA, US) fibre ablation (2013 nm, 15W) and for the parathyroidectomy, an ultrasonic Harmonic Ace Curved Dissector (Ethicon Endo-surgery, JnJ, US) was used (3–5W). For real-time tissue classification a main recognition window was displayed on the instrument’s monitor. The main output included a classification result (which can be colour according to the specific tissue type etc.), a real time TIC as well as a probability number, which is based on the class distances within the discriminant analysis model. At this point the surgeon is blinded to any mass spectra information and classification results as well as the output is not yet used for clinical decision making due to regulatory reasons (the system can be used for research purposes only).

### Statistical analysis and spectral interpretation

Raw spectral data were processed in Abstract Model Builder (AMX) (v. 0.9.2092 Waters Research Centre, Hungary), having undergone background subtraction, normalization, and lock mass correction using Leucine Enkephalin at *m/z* 554.2615. For Fig. 3, the representative spectra underwent lock mass correction using Leucine Enkephalin although the *m/z* 554.2615 is not shown (full spectra is shown in Fig. S3). Binning was performed at 0.1 and the spectra were generated using one spectrum per scan. Spectral interpretation and processing were done in MassLynx (v.4.2, Waters Corp., UK). For background subtraction, a locally adaptive model of noise was used (MassLynx v.4.2), which adjusts the zero level in the continuum spectrum to lessen the effect of chemical noise. For calculating the TIC, signal intensities, and background noise values all data matrices were imported into the Spectrum_Quality_Analyzer (Waters Research Centre, Hungary), where subgroup analyses was performed comparing the sum of the most 20 intensive peaks with the mean of the whole spectrum to identify an optimal cut-off for inclusion..Low-quality spectra were excluded before modelling, based on pre-defined criteria: for palate signal:noise ratio < 900, 28 spectra were included out of a total of 64 spectra, for tongue signal:noise ratio < 400, 18 spectra were included out of a total of 28 spectra, and for epiglottis all spectra were included. A summary of all data values is included in the Supplementary Information, Table S9. During the analysis, and specifically for the palate, all the initial ablation points of the target tissue were included (scans 198–262) where fresh tissue was analysed while the previously energized points/scans where not included as no biological signal was observed in the resampling points. For finding the bins with the highest relative abundance in the m/z range of 560–1000, the total mean of each bin was selected and calculated from the raw data. The observed masses were matched against the theoretical masses using ChemCalc^34^. Putative identification of compounds was done using LIPID MAPS^35^. Multivariate statistical analysis and spectral classification were performed using PCA and OPLS-DA in SIMCA (v. 16.1, Umetrics, Sweden). The validity and the degree of overfit for the OPLS-DA model was assessed using permutation randomisation tests. For the permutation plots n = 999 repetitions were used. For multivariate modelling in the TORS cases, the m/z range of 560–1000 was used, where lipid metabolites can be utilized for tissue identification in a robust manner, i.e. the contribution of background and contamination is minimal. Univariate statistical analysis (ANOVA) was performed in R Studio (v1.1.419, R Core Team, Vienna, Austria) to identify statistically significant ions which separated the groups defining a cut-off of *p* < 0.05 using the False Discovery Rate (FDR) method. For pairwise comparisons between the different sampling points, the p values were determined using a two-tailed t-Test with unequal variances. For visualization of the OPLS-DA predictive component, S-plots were made in SIMCA (v. 16.1, Umetrics, Sweden).

### Supplementary Information

Supplementary Information 1.

Supplementary Video S1.

Supplementary Video S2.

## Data Availability

The data that support the findings of this study are available from the corresponding author upon reasonable request.
