# Supplementary material for: Human robotic surgery with intraoperative tissue identification using rapid evaporation ionisation mass spectrometry
Source: Sci Rep. 2024 Jan 10;14:1027. doi: 10.1038/s41598-023-50942-3 (PMC10781715; doi:10.1038/s41598-023-50942-3)
Supplement: Supplementary file 1 — Supplementary Information 1. [file 41598_2023_50942_MOESM1_ESM.docx]

**Supplementary information**

First in Human Robotic Surgery with Intraoperative Tissue Identification using Rapid Evaporation Ionisation Mass Spectrometry

**Eftychios Manoli^1^, James Higginson^1^, Neil Tolley^2^, Ara Darzi^2^, James Kinross^2^, Burak Temelkuran^1,3+^, Zoltan Takats^1*+^**

^1^ Department of Metabolism, Digestion and Reproduction, Imperial College London, London, UK

^2^ Department of Surgery and Cancer, Imperial College London, London, UK

^3^ The Hamlyn Centre for Robotic Surgery, Imperial College London, London, UK

^+^ These authors contributed equally: Burak Temelkuran, Zoltan Takats

^*^ ^Corresponding author: Correspondence to Zoltan Takats^

**Table of contents**

**Supplementary text (S1-S2) 2**

**Supplementary figures (S1-S9) 5**

**Supplementary tables (S1-S8) 15**

**Supplementary movie legends (S1-S2) 27**

**References 28**

**Supplementary Text S1**

**REIMS Interface Optimisation**

Two different methods of transferring the nascent surgical aerosol into the MS instrument were tested *in vivo*. The first one utilized the Venturi air jet pump driven by medical air (2 bar) as part of the REIMS set-up (Figure S1a(i)) and the second method used the direct introduction of aerosol via a T piece connector (Figure S1a(ii)). Each method uses a different mechanism of transferring the surgical aerosol from the point of ablation/dissection to the MS instrument, which appears to affect the signal generated by REIMS. Table S1 shows how each method impacts different REIMS spectra characteristics including the Total Ion Count (TIC), signal intensity, background noise, and signal-to-noise ratio, during the laser ablation of the palate in the *m/z* range of 600-1000. TIC measured using the Venturi method was significantly lower (by 84.2%) than the TIC measured with the direct introduction method (*p*=0.00004, Table S1). Mean signal intensity obtained from the Venturi method was also lower by 60% (0.19 to 0.07, Table S1) along with a considerable increase by 87% in background noise level. This caused a 79% reduction in signal-to-noise when the Venturi method was used compared to the direct introduction method (*p* = 0.002, Table S1).

The spectra of the 20 ions with the highest relative abundance in the *m/z* range of 600-1000 showed distinct differences between the Venturi and the direct introduction method (Figure S1b & c). It was evident that less biological signal is being generated using the Venturi method, with 50% of the ions observed representing background noise peaks with the rest of the ions (50%) being tissue-specific lipid metabolites (Table S2). In contrast, using the direct introduction method, no background noise peaks were found, with 75% of the ions observed representing lipid metabolites with the remaining 25% of the ions corresponding to metabolites yet to be identified (Table S3).

Between both methods, a variety of diglycerides and ceramides were observed and putatively identified using LIPID MAPS, with all the metabolites being ionized with a chloride adduct, except for a single ion which was deprotonated (*m/z* at 750.588). The differences in spectra can be also seen in the PCA plot (Figure S2a) where there is clear clustering by the different method used and in an OPLS-DA plot (Figure S2b), which has a high predictive ability with R^2^X= 0.99 and Q^2^=0.96.

Spectral similarities were also observed, with 40% of the 20 most intense peaks (including isotopes) identified in the *m/z* range of 600-1000 being present in the spectra achieved from both methods (Figure S1d). These include mostly diglycerides and ceramides and they are all found to be statistically significantly higher in abundance in the spectra achieved using the direct introduction method (Figure S1d).

The signal observed using the Venturi setup is constituted by a mixture of high background noise peaks along with low abundant lipid metabolites. This general reduction in the relative abundance of each true peak and the considerable increase of the peaks constituting background noise can be explained by the fact that the Venturi effect creates drag of flow of surgical aerosol around 2 L/min, which translates to an effective 30 times dilution of the sample. On the other hand, the direct introduction method using the T piece connector creates a flow of around 750 mL/min through the inlet capillary (0.5 mm internal diameter). In this approach the dilution effect is minimal, resulting in better quality REIMS spectra where the contribution of background is low and lipid metabolites are easily identifiable (Figure S1c and Table S2). In addition, the Venturi setup is less sensitive to aspirated liquids as it creates a fine mist of droplets which is undesirable for this technique (will disable the MS), compared to the direct aspiration. When using the Venturi pump, the REIMS-based instrument provided a < 2 s time delay between surgical ablation/dissection and instrumental feedback. In the case of the direct aspiration method, a < 3 s time delay was observed. Despite that, the direct introduction method was chosen as an optimal method for these feasibility studies, as it produced better quality spectra, where the background noise level is low, and a high number of lipid metabolites are easily identifiable.

Regardless of choosing the direct introduction method as the optimal smoke evacuation set-up for this study, both methods can be efficiently adapted with the REIMS interface, depending on the clinical application and the amount/concentration of surgical aerosol produced ^1-3^.

**Supplementary Text 2**

The implementation of various energy devices to REIMS involves different methods of heating, producing a different composition of the aerosol particles. The thulium laser used in the TORS cases presents various benefits in tissue manipulation, diagnostics (coupled to REIMS) as well as robotic integration for precision surgery. The wavelength of the laser plays an important role in the way the laser energy interacts with the target tissue, affecting the surgical precision and the REIMS signal. In the UV – VIS range, up to green light wavelengths (180 nm – 530 nm), tissue constituents such as protein (with absorption peak around 190 nm), haemoglobin (present in vascular tissue), and melanin (present in pigmented tissue like skin) are active absorbing elements and contribute significantly to the absorption of light via electronic excitation of molecules (Figure S9)^4, 5^. From the red-light wavelengths up to the NIR range (650 nm to 1200 nm), the overall absorption of tissue constituents drops, increasing the penetration depth of the light into the tissue, and scattering becomes the dominant light-tissue interaction. While this interaction is very helpful in controlling bleeding, it is a limiting factor for surgical precision. The interaction of light with tissue in the Near–Mid IR is mostly dominated by the vibrational modes of the water molecules and their combinations, resulting in several absorption peaks shown in Figure S9. Resonance absorption of collagen molecules also contributes to the interaction in the range of 6 - 8 microns spectral range. When tissue is exposed to a laser beam at a water resonant absorption wavelength, the energy is initially absorbed by only the water molecules. Thanks to the high absorption coefficient at these resonances, all the energy is absorbed rapidly by the water molecules in a small volume close to the surface, resulting in rapid evaporation of the tissue, preventing fragmentation of molecules of interest, and facilitating the ionization of molecules necessary for the REIMS system^6^. In addition, the shallow penetration depth of the laser beam results in low thermal damage to the surrounding tissue, improving the precision of the surgery. The development of specialised flexible delivery mechanisms (hollow-core fibres) designed for these wavelengths^7, 8^, enabled the use of the CO_2_ laser (10.6 µm) in precision robotic surgery^9^.

The wavelength of the laser used in this study (2 µm) is located around one of the strong absorption bands of OH groups in water^10^ but with an absorption coefficient an order of magnitude smaller in amplitude than that of the CO_2_ laser. While presenting surgical precision provided by the resonant absorption of water molecules, the relatively deep penetration of the thulium laser allows excellent bleeding control and makes this laser a very good candidate for robotic surgery^11^, an important benefit when compared to the limited microvascular coagulation capacity of the CO_2_ laser. Another benefit of this wavelength is its delivery mechanism. Unlike the Mid-IR wavelengths requiring special hollow-core fibres, this wavelength can be transmitted through conventional cost-efficient silica fibres.

In the TORS cases, various glycerophospholipids were observed because of the light-induced thermal evaporation seen by the thulium surgical laser. In contrast, during the parathyroidectomy case, the ion formation mechanism is based on the combination of heat and mechanical vibration on tissue, where phospholipid bilayers are not destroyed, and the generated surgical aerosol is formed of interstitial fluid mostly containing non-membrane lipids (such as diglycerides and triglycerides) as previously reported. Despite the fact that different ionisation mechanisms are taking place tissue specificity is retained.

**Supplementary Figures**

**
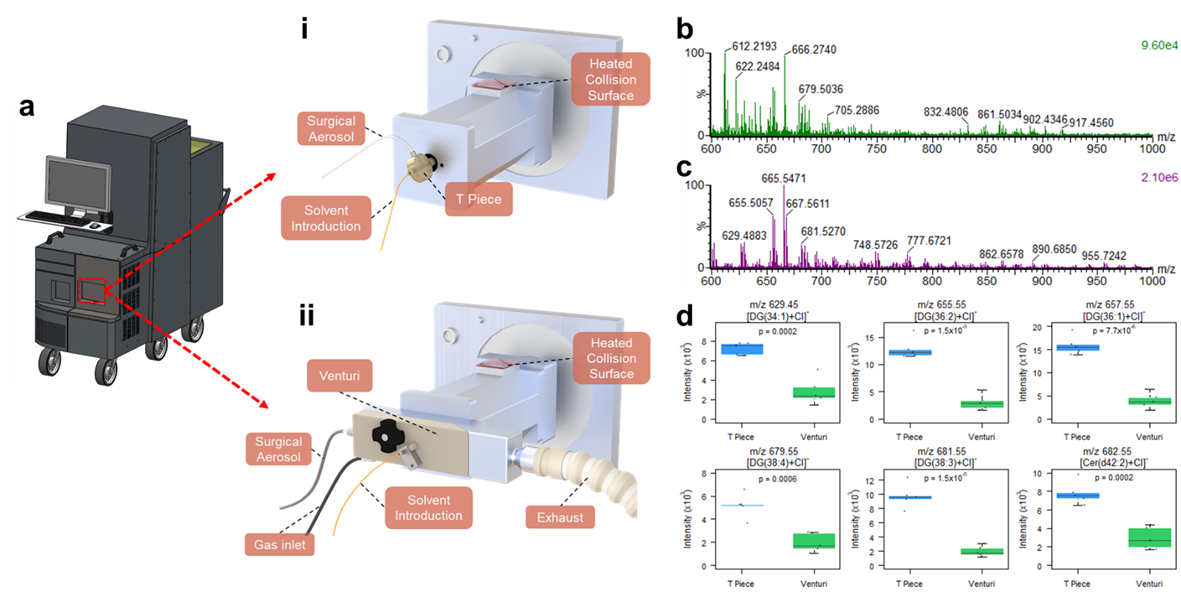
**

**Figure S1. REIMS surgical aerosol transferring setups and spectra interpretation**

**(a)** Schematic representation of the REIMS interface equipped with i) the Venturi air pump and ii) direct introduction method using the T piece connector. Average REIMS spectra using **(b)** the direct introduction method **(c)** the Venturi pump, during the laser ablation of the palate in the *m/z* range of 600-1000. Background subtraction and lockmass correction (at *m/z* 554.2615) were applied. **(d)** Box plots showing the intensity of common lipid ions identified during the REIMS interface optimization using the Venturi pump and the direct introduction method. The box represents the interquartile range with the median shown. The whiskers represent the range of data points. Raw data are represented using jitter points. ANOVA was performed (R Studio) between the two groups using the False Discovery Rate (FDR) method for *p*-value correction.


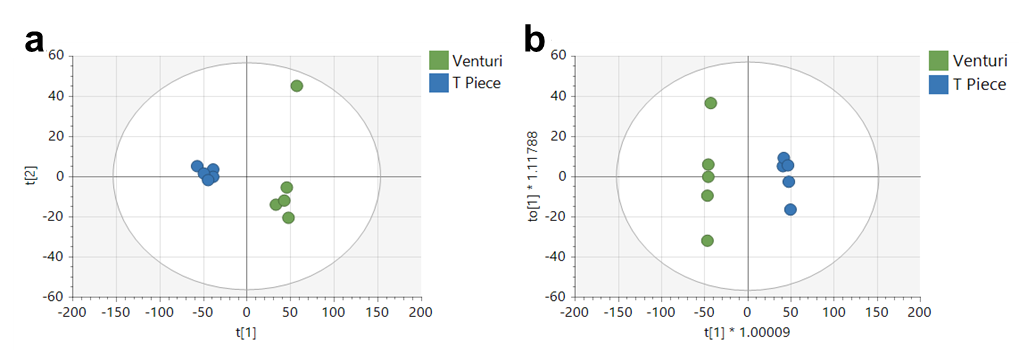


**Figure S2. Multivariate statistical analysis of selected spectra generated using the Venturi pump and the direct introduction method.**

**(a)** PCA plot of the Venturi and direct introduction method in the *m/z* range of 600-1000. The first two components were most responsible for the variance observed with PC1=58.7% and PC2=7.9%. No outliers were observed based on the ellipse Hotelling’s T2. **(b)** OPLS-DA plot of the two methods in the *m/z* range of 600-1000. One predictive and one orthogonal component showing high predictive ability with R^2^X= 0.99 and Q^2^=0.96.

**
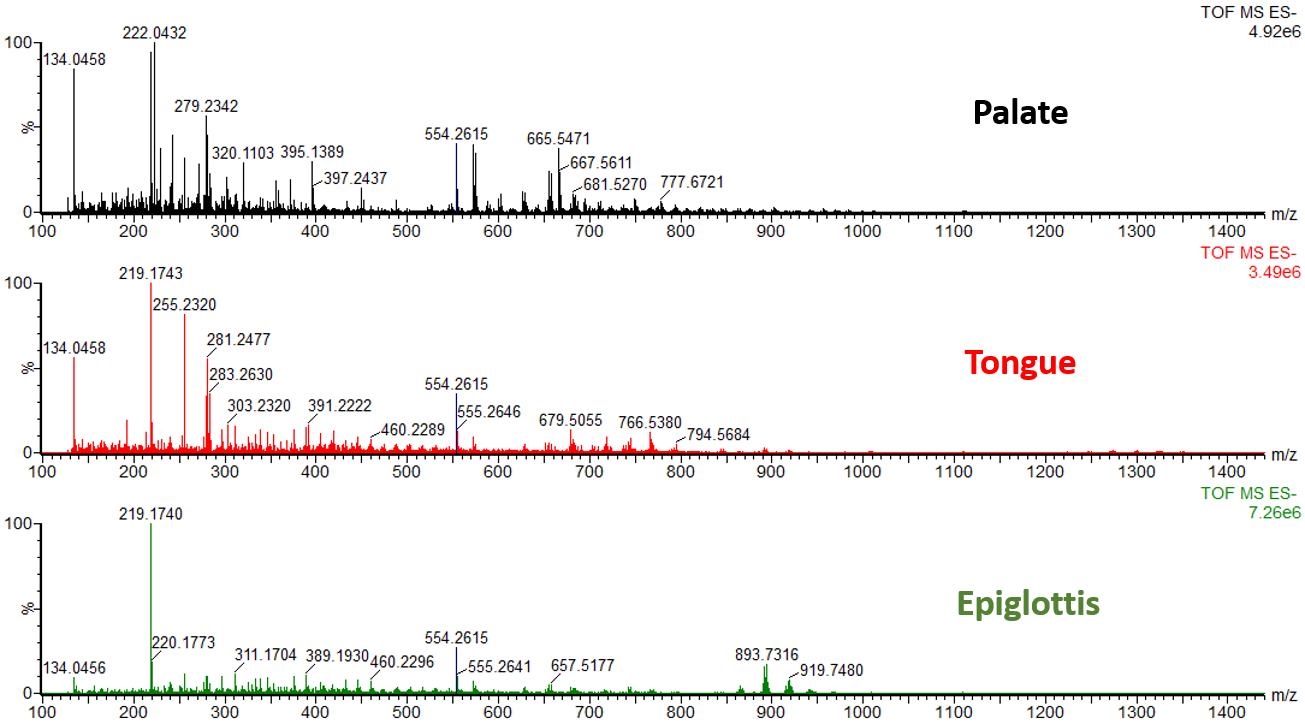
**

**Figure S3. Mass spectra acquired on different tissues during the iKnife-TORS cases.**

Averaged mass spectra acquired during the ablation of the palatal NKSSE (black), lingual KSSE (red), and epiglottic NKSSE (green). Data were acquired in the negative mode in the *m/z* range of 100-1500 using a Xevo G2-S instrument (Waters Corporation, UK). The y-axis represents the relative abundance and the x-axis the mass-to-charge ratio of ions. Spectra were processed in MassLynx (v.4.2, Waters Corp., UK) where background subtraction was applied. Lockmass correction was applied using Leucine Enkephalin at *m/z* 554.2615. The peak at *m/z* 219.17 appears to be dominant in all the spectra (and it was observed in the background when the propa-2-ol solvent solution was used) and is yet to be identified.


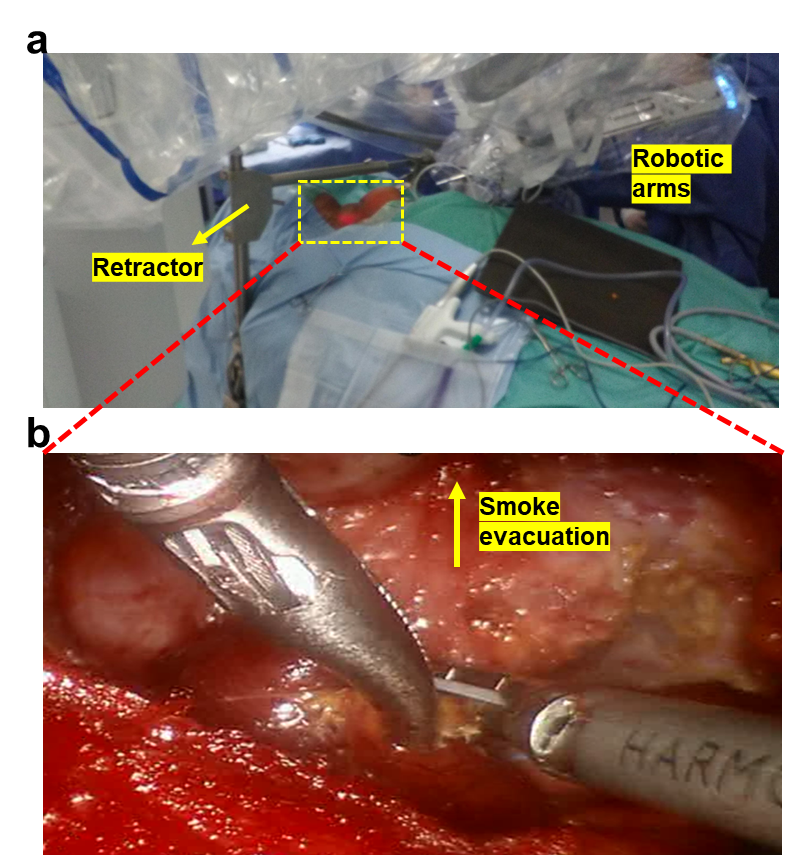


**Figure S4. iKnife - Parathyroidectomy intraoperative set-up.**

**(a)** Left superior transaxillary parathyroidectomy set-up. A special retractor was used to open the space for the robotic arms and was mounted on the other side of the patient’s table. The REIMS suction tube was placed inside and underneath the retractor, facilitating continuous aspiration of the generated plumes. **(b)** Surgeon’s view of the removal of the parathyroid gland. The 5mm long tip Maryland dissector (left) and the Harmonic Ace Curved Shears (right) were used for tissue manipulation and dissection. The smoke evacuation was achieved from the top (yellow arrow) of the anterior axilla by placing the aspiration tube inside and underneath the retractor.


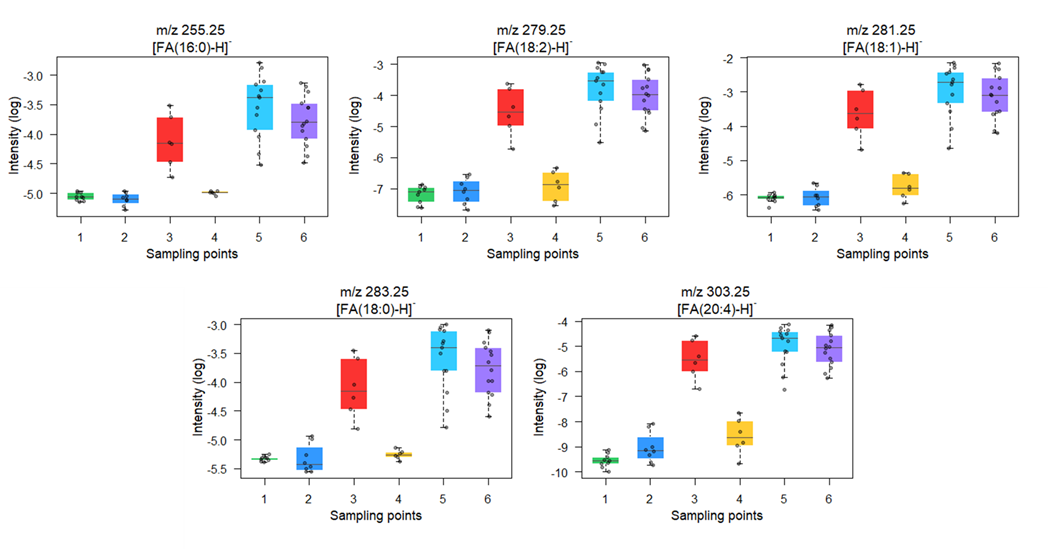


| m/z | Sampling points | *p-value* | Sampling points | *p-value* | Sampling points | *p-value* | Sampling points | *p-value* | Sampling points | *p-value* |
| --- | --- | --- | --- | --- | --- | --- | --- | --- | --- | --- |
| 255.25 | 1 & 2 | 0.38 | 2 & 3 | **0.02** | 3 & 4 | **0.02** | 4 & 5 | **6.5E-05** | 5 & 6 | 0.15 |
|  | 1 & 3 | **0.02** | 2 & 4 | **0.03** | 3 & 5 | **0.01** | 4 & 6 | **1.6E-05** |  |  |
|  | 1 & 4 | **0.03** | 2 & 5 | **5.1E-05** | 3 & 6 | 0.10 |  |  |  |  |
|  | 1 & 5 | **5.6E-05** | 2 & 6 | **1.0E-05** |  |  |  |  |  |  |
|  | 1 & 6 | **1.2E-05** |  |  |  |  |  |  |  |  |
|  |  |  |  |  |  |  |  |  |  |  |
| 279.25 | 1 & 2 | 0.41 | 2 & 3 | **0.02** | 3 & 4 | **0.02** | 4 & 5 | **4.8E-05** | 5 & 6 | 0.29 |
|  | 1 & 3 | **0.02** | 2 & 4 | 0.44 | 3 & 5 | **0.02** | 4 & 6 | **7.5E-05** |  |  |
|  | 1 & 4 | 0.19 | 2 & 5 | **4.6E-05** | 3 & 6 | 0.12 |  |  |  |  |
|  | 1 & 5 | **4.4E-05** | 2 & 6 | **6.9E-05** |  |  |  |  |  |  |
|  | 1 & 6 | **6.6E-05** |  |  |  |  |  |  |  |  |
|  |  |  |  |  |  |  |  |  |  |  |
| 281.25 | 1 & 2 | 0.65 | 2 & 3 | **0.02** | 3 & 4 | **0.02** | 4 & 5 | **4.2E-05** | 5 & 6 | 0.39 |
|  | 1 & 3 | **0.02** | 2 & 4 | 0.13 | 3 & 5 | **0.02** | 4 & 6 | **7.1E-05** |  |  |
|  | 1 & 4 | 0.08 | 2 & 5 | **3.7E-05** | 3 & 6 | 0.11 |  |  |  |  |
|  | 1 & 5 | **3.6E-05** | 2 & 6 | **6.0E-05** |  |  |  |  |  |  |
|  | 1 & 6 | **5.9E-05** |  |  |  |  |  |  |  |  |
|  |  |  |  |  |  |  |  |  |  |  |
| 283.25 | 1 & 2 | 0.84 | 2 & 3 | **0.02** | 3 & 4 | **0.02** | 4 & 5 | **2.6E-05** | 5 & 6 | 0.28 |
|  | 1 & 3 | **0.02** | 2 & 4 | 0.62 | 3 & 5 | **0.04** | 4 & 6 | **1.5E-05** |  |  |
|  | 1 & 4 | 0.10 | 2 & 5 | **2.2E-05** | 3 & 6 | 0.17 |  |  |  |  |
|  | 1 & 5 | **2.3E-05** | 2 & 6 | **1.2E-05** |  |  |  |  |  |  |
|  | 1 & 6 | **1.3E-05** |  |  |  |  |  |  |  |  |
|  |  |  |  |  |  |  |  |  |  |  |
| 303.25 | 1 & 2 | 0.08 | 2 & 3 | **0.02** | 3 & 4 | **0.02** | 4 & 5 | **3.7E-05** | 5 & 6 | 0.45 |
|  | 1 & 3 | **0.02** | 2 & 4 | 0.25 | 3 & 5 | 0.09 | 4 & 6 | **5.8E-05** |  |  |
|  | 1 & 4 | 0.05 | 2 & 5 | **3.4E-05** | 3 & 6 | 0.26 |  |  |  |  |
|  | 1 & 5 | **3.2E-05** | 2 & 6 | **5.2E-05** |  |  |  |  |  |  |
|  | 1 & 6 | **4.8E-05** |  |  |  |  |  |  |  |  |

**Figure S5. Box plots and pairwise comparison of statistically significant fatty acids during the iKnife-parathyroidectomy case.** The box plots are showing the log scale intensity of different fatty acid ions identified for all six sampling events. A characteristic increase in signal intensity was observed in sampling points 3,5 and 6 compared to sampling points 1,2 and 4. The box represents the interquartile range with the median shown. The whiskers represent the range of data points. Raw data are represented using jitter points. ANOVA was performed (R Studio) between the different sampling points using the FDR method and defining a cut-off of *p* <0.05. Pairwise comparison between all possible combinations for all six sampling points was done. *p* values were determined using a two-tailed t-Test with statistically significant findings shown in bold and underlined.


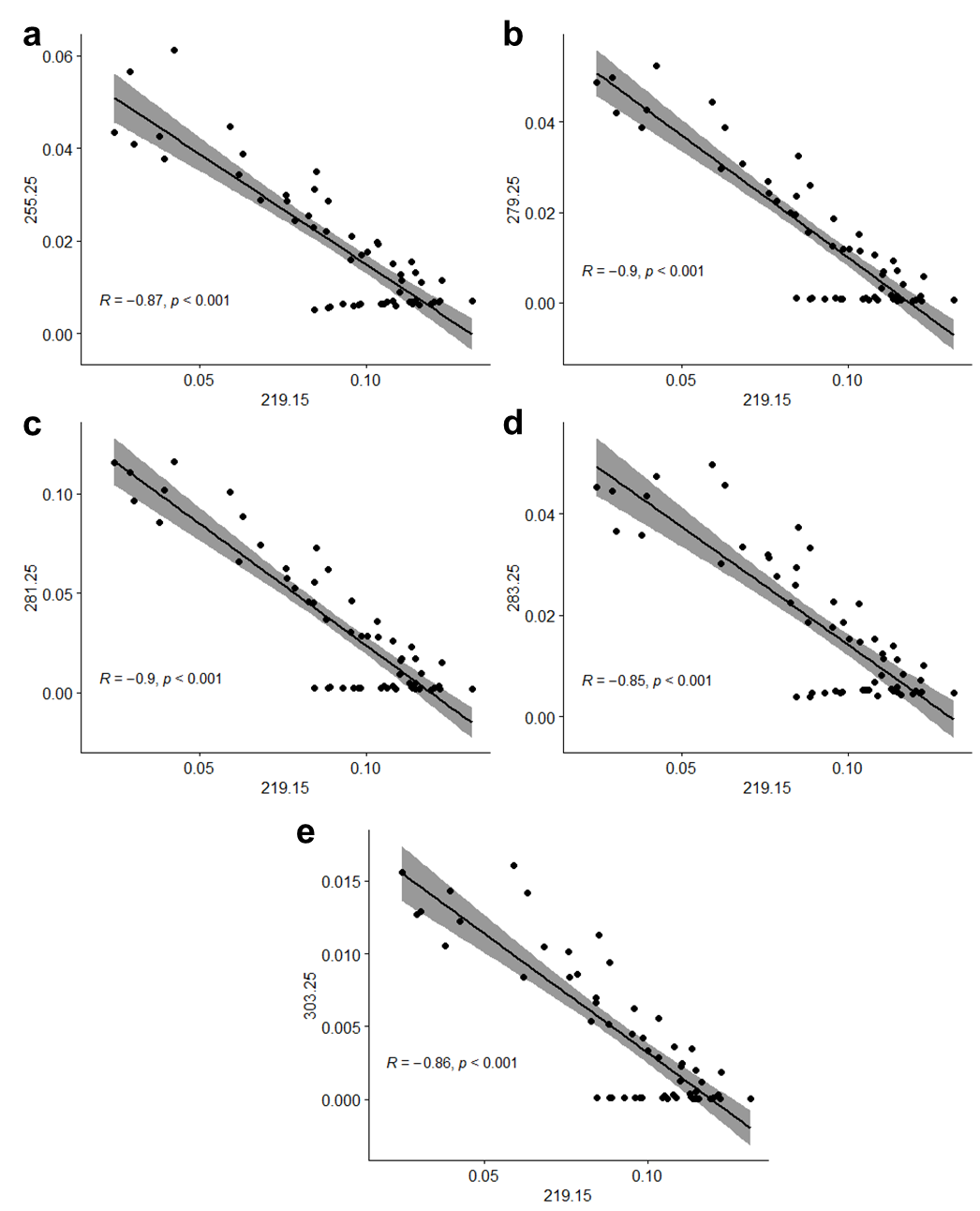


**Figure S6. Pearson correlation analysis of the signal intensities between the *m/z* 219.15 with various fatty acids during the iKnife-parathyroidectomy case. (a-e)** The y-axis of the plots represents the various *m/z* of the fatty acids and the x-axis the *m/z* of 219.15. For all scatter plots the high values of the fatty acids (y-axis) are associated with low levels of the ion at 219.15 (x-axis). Scatter plots show a strong linear negative correlation with correlation coefficients (R) ranging between -0.85 and -0.9. All correlations were statistically significant with *p*<0.001.


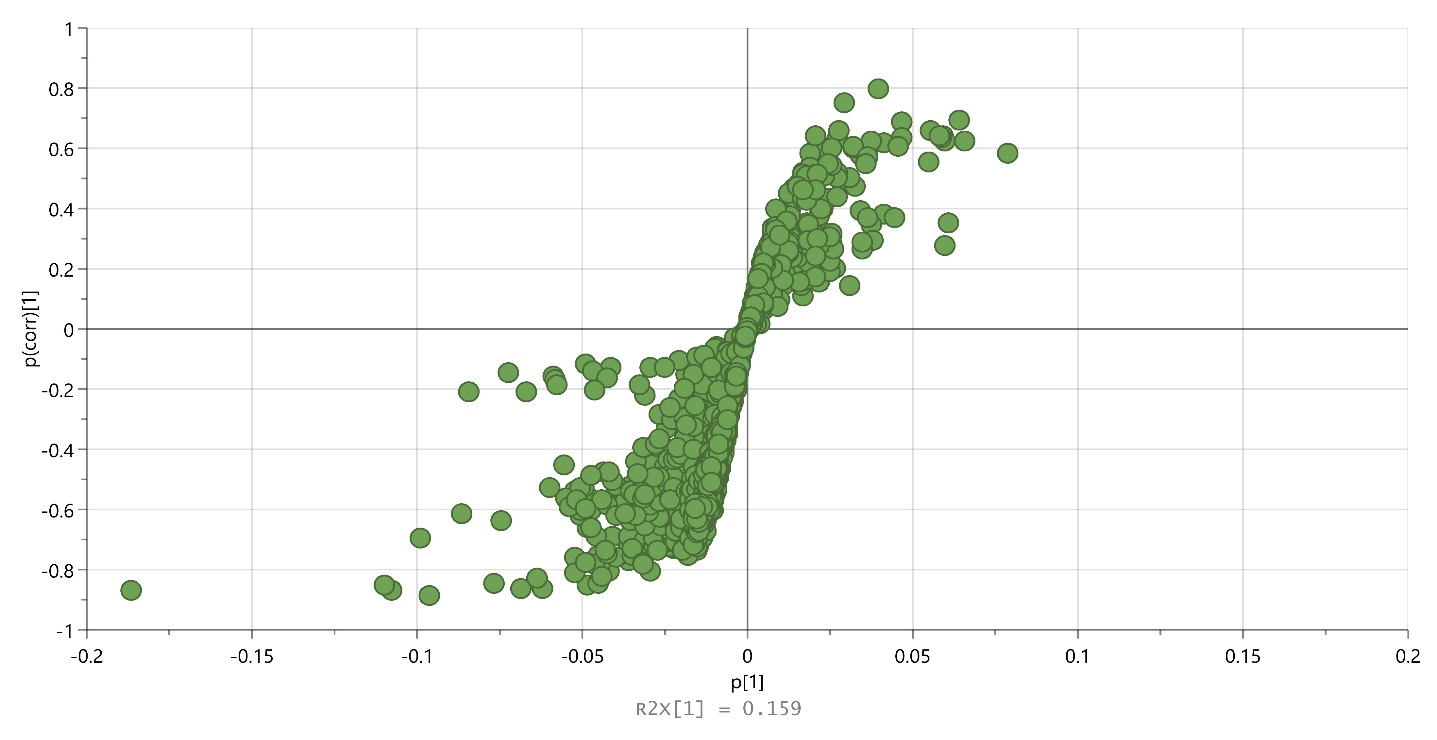


**a**

**722.55**

**875.75**

**612.25**


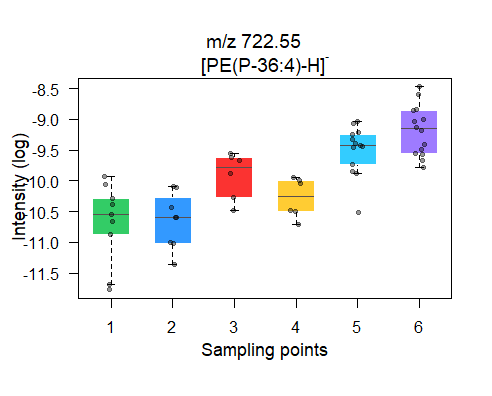

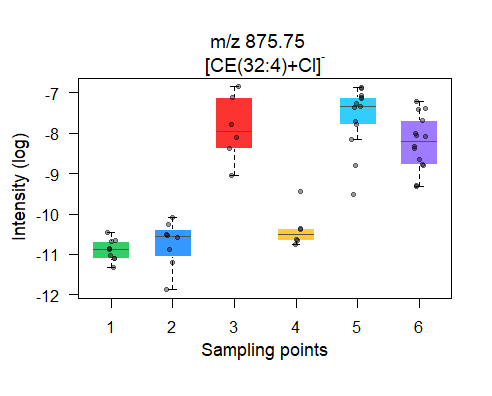


**b**

**c**

**Figure S7. Statistical analysis of sampling points in the *m/z* range of 600-1000 during the iKnife-parathyroidectomy case. (a)** S-plot analysis of the different sampling points associated with thyroid and parathyroid in the *m/z* range of 600-1000 using Pareto scaling. The axes that are plotted in the S-plot from the predictive component are p1 vs p(corr)1, representing the magnitude (modelled covariation) and reliability (modelled correlation) respectively^12^. Here, the ion at *m/z* 722.55 (*p*=3.18E^-10^) shows very high reliability where the ion at *m/z* 875.75 (*p*=0.0002) has a high model influence because of the high variance on the dataset. The ion at *m/z* 612.25 shows a very low magnitude and reliability and is yet to be identified. ANOVA was performed (R Studio) between the different sampling points using the FDR method (*p* <0.05). **(b-c)** Box plots showing the log scale intensity of ions at *m/z* 722.55 and *m/z* 875.75 in all the sampling points. The box represents the interquartile range with the median shown. The whiskers represent the range of data points. Raw data are represented using jitter points. (*PE-Phosphatidylethanolamine, CE-Cholesteryl ester*).


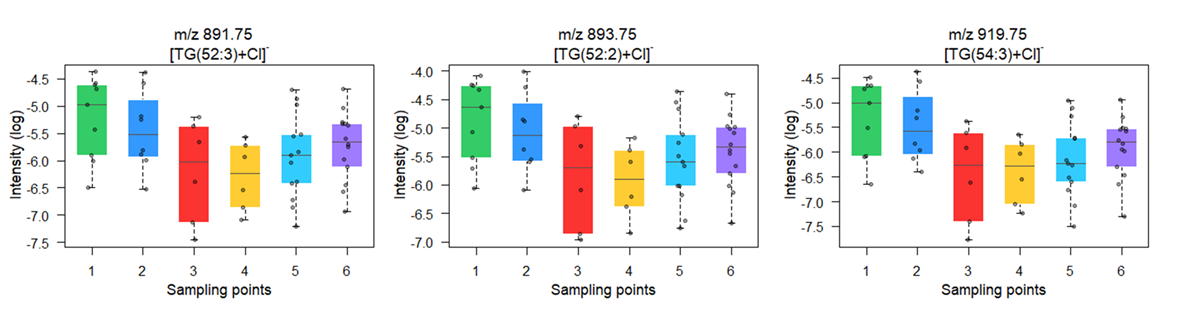


| m/z | Sampling points | *p-value* | Sampling points | *p-value* | Sampling points | *p-value* | Sampling points | *p-value* | Sampling points | *p-value* |
| --- | --- | --- | --- | --- | --- | --- | --- | --- | --- | --- |
| 891.75 | 1 & 2 | 0.53 | 2 & 3 | 0.14 | 3 & 4 | 0.55 | 4 & 5 | 0.16 | 5 & 6 | 0.73 |
|  | 1 & 3 | **0.03** | 2 & 4 | 0.06 | 3 & 5 | 0.53 | 4 & 6 | 0.05 |  |  |
|  | 1 & 4 | **0.01** | 2 & 5 | 0.26 | 3 & 6 | 0.32 |  |  |  |  |
|  | 1 & 5 | 0.06 | 2 & 6 | 0.33 |  |  |  |  |  |  |
|  | 1 & 6 | 0.08 |  |  |  |  |  |  |  |  |
|  |  |  |  |  |  |  |  |  |  |  |
| 893.75 | 1 & 2 | 0.55 | 2 & 3 | 0.15 | 3 & 4 | 0.54 | 4 & 5 | 0.07 | 5 & 6 | 0.83 |
|  | 1 & 3 | **0.03** | 2 & 4 | 0.06 | 3 & 5 | 0.59 | 4 & 6 | 0.07 |  |  |
|  | 1 & 4 | **0.01** | 2 & 5 | 0.24 | 3 & 6 | 0.45 |  |  |  |  |
|  | 1 & 5 | 0.06 | 2 & 6 | 0.27 |  |  |  |  |  |  |
|  | 1 & 6 | 0.06 |  |  |  |  |  |  |  |  |
|  |  |  |  |  |  |  |  |  |  |  |
| 919.75 | 1 & 2 | 0.66 | 2 & 3 | 0.08 | 3 & 4 | 0.77 | 4 & 5 | 0.29 | 5 & 6 | 0.60 |
|  | 1 & 3 | **0.02** | 2 & 4 | 0.05 | 3 & 5 | 0.55 | 4 & 6 | 0.09 |  |  |
|  | 1 & 4 | **0.01** | 2 & 5 | 0.13 | 3 & 6 | 0.29 |  |  |  |  |
|  | 1 & 5 | **0.03** | 2 & 6 | 0.18 |  |  |  |  |  |  |
|  | 1 & 6 | 0.05 |  |  |  |  |  |  |  |  |
|  |  |  |  |  |  |  |  |  |  |  |

**Figure S8. Box plots and pairwise comparison of statistically significant triglycerides in the *m/z* range of 800-1000 during the iKnife-parathyroidectomy case.** The box plots are showing the log scale intensity of different triglyceride ions identified for all six sampling events. The box represents the interquartile range with the median shown. The whiskers represent the range of data points. Raw data are represented using jitter points. ANOVA was performed between the different sampling points using the FDR method for calculating *p* values (*m/z* at 891.7197 *p*=0.023, *m/z* at 893.7324 *p*=0.014, and *m/z* at 919.7485 *p*=0.0037). Pairwise comparison between all possible combinations for all six sampling points was done. *p* values were determined using a two-tailed t-Test with statistically significant findings shown in bold and underlined. For all ions, statistical significance was observed between sampling points 1&3 and 1&4. In addition, for ion at *m/z* 919.75 statistical significance was found between sampling points 1&5. The other combinations between sampling points for each ion show no statistical significance with p>0.05.


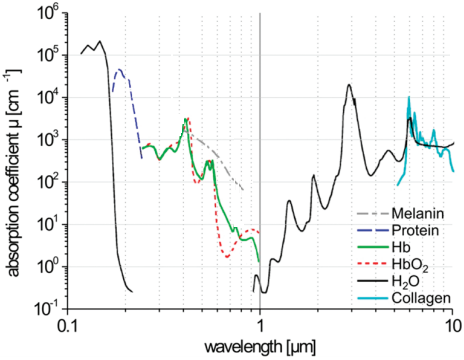


**Figure S9. Generalized electromagnetic absorbance spectrum of mammalian tissues**^4, 5^ (Reproduced with permission from Supporting Reference 8, Copyright 2003 American Chemical Society)

**Supplementary Tables**

**Table S1. Details of Venturi and direct introduction methods, on how they impact various spectral characteristics in the *m/z* range of 600-1000.** ‘Signal’ is defined as the total intensity of the 20 highest peaks in the different *m/z* ranges and ‘Noise’ is defined as the median intensity of peaks within each *m/z* range. For pairwise comparisons p values were determined using a two tailed t-Test with statistically significant findings shown in bold.

| m/z range | Variables | Spectra | Mean TIC x10^7^ (range) | *P-value* | Mean Signal x10 (range) | Mean Noise x10^-5^ (range) | Mean SNR x10^3^ (range) | *P-value* |
| --- | --- | --- | --- | --- | --- | --- | --- | --- |
| 600-1000 | Venturi | 5 | 0.24 (0.22-0.26) | **0.00004** | 0.76 (0.66-0.86) | 14.26 (13-15.6) | 0.54 (0.42-6.63) | **0.002** |
|  | Direct introduction (T piece) | 5 | 1.52 (1.37-1.77) |  | 1.92 (1.65-2.36) | 7.59 (6.36-8.39) | 2.59 (2.10-3.72) |  |

**Table S2. The 20 ions with the highest intensity identified in the *m/z* range of 600-1000 using the Venturi setup**. The ions were putatively identified using accurate masses on LIPID MAPS with the ppm error <5 *(DG-Diglycerides, Cer-Ceramides).*

| No | Bin | Observed mass | Theoretical mass | Relative abundance x10^3^ | Identity | Adduct | ppm error |
| --- | --- | --- | --- | --- | --- | --- | --- |
| 1 | 611.25 | 611.2815 | - | 5.47 | Background noise | - | - |
| 2 | 612.25 | 612.2215 | - | 8.89 | Unknown | - | - |
| 3 | 613.25 | 613.2156 | - | 3.70 | Background noise | - | - |
| 4 | 614.25 | 614.2167 | - | 3.19 | Background noise | - | - |
| 5 | 622.25 | 622.248 | - | 5.32 | Background noise | - | - |
| 6 | 627.45 | 627.475 | 627.4761 | 3.42 | DG(34:2) | [M+Cl]- | -1.75 |
| 7 | 629.45 | 629.4888 | 629.4911 | 5.04 | DG(34:1) | [M+Cl]- | -3.65 |
| 8 | 639.25 | 639.2355 | - | 3.10 | Background noise | - | - |
| 9 | 644.25 | 644.232 | - | 2.77 | Background noise | - | - |
| 10 | 653.45 | 653.4886 | 653.4911 | 3.67 | DG(36:3) | [M+Cl]- | -3.82 |
| 11 | 655.55 | 655.5052 | 655.5068 | 5.32 | DG(36:2) | [M+Cl]- | -2.44 |
| 12 | 656.55 | 656.5085 | 656.5102 | 3.44 | ^13^C isotope of DG(36:2) | [M+Cl]- | -2.58 |
| 13 | 657.55 | 657.5198 | 657.5224 | 6.34 | DG(36:1) | [M+Cl]- | -3.95 |
| 14 | 658.55 | 658.5228 | 658.5258 | 2.62 | ^13^C isotope of DG(36:1) | [M+Cl]- | -4.56 |
| 15 | 666.25 | 666.2755 | - | 7.39 | Background noise | - | - |
| 16 | 667.25 | 667.275 | - | 3.06 | Background noise | - | - |
| 17 | 679.55 | 679.5045 | 679.5068 | 2.87 | DG(38:4) | [M+Cl]- | -3.38 |
| 18 | 681.55 | 681.5205 | 681.5224 | 3.03 | DG(38:3) | [M+Cl]- | -2.78 |
| 19 | 682.55 | 682.5902 | 682.5905 | 4.39 | Cer(d42:2) | [M+Cl]- | -0.43 |
| 20 | 688.25 | 688.2557 | - | 2.79 | Background noise | - | - |

**Table S3. The 20 ions with the highest intensity identified in the *m/z* range of 600-1000 using the direct introduction method**. The ions were putatively identified using accurate masses on LIPID MAPS with the ppm error <5 *(Cer-Ceramides, DG-Diglycerides, HexCer-Hexosyl ceramides).*

| No | Bin | Observed mass | Theoretical mass | Relative abundance x10^3^ | Identity | Adduct | ppm error |
| --- | --- | --- | --- | --- | --- | --- | --- |
| 1 | 626.55 | 626.5302 | 626.5279 | 13.37 | Cer(d38:2) | [M+Cl]- | 3.67 |
| 2 | 628.55 | 628.5462 | 628.5435 | 9.67 | Cer(d38:1) | [M+Cl]- | 4.29 |
| 3 | 629.45 | 629.4892 | 629.4911 | 7.76 | DG(34:1) | [M+Cl]- | -3.01 |
| 4 | 655.55 | 655.507 | 655.5068 | 16.13 | DG(36:2) | [M+Cl]- | -0.30 |
| 5 | 656.55 | 656.5083 | 656.5102 | 11.03 | ^13^C isotope of DG(36:2) | [M+Cl]- | -2.89 |
| 6 | 657.55 | 657.5198 | 657.5224 | 19.19 | DG(36:1) | [M+Cl]- | -3.95 |
| 7 | 658.55 | 658.5226 | 658.5258 | 8.94 | ^13^C isotope of DG(36:1) | [M+Cl]- | -4.86 |
| 8 | 665.55 | 665.5475 | - | 34.71 | Unknown | - | - |
| 9 | 666.55 | 666.5515 | - | 15.41 | Unknown | - | - |
| 10 | 667.55 | 667.5621 | - | 19.97 | Unknown | - | - |
| 11 | 668.55 | 668.577 | 668.5748 | 8.45 | Cer(d41:2) | [M+Cl]- | 3.29 |
| 12 | 679.55 | 679.5065 | 679.5068 | 6.56 | DG(38:4) | [M+Cl]- | -0.44 |
| 13 | 681.55 | 681.525 | 681.5224 | 12.36 | DG(38:3) | [M+Cl]- | 3.81 |
| 14 | 682.55 | 682.5883 | 682.5905 | 9.78 | Cer(d42:2) | [M+Cl]- | -3.22 |
| 15 | 683.55 | 683.5413 | 683.5381 | 9.80 | DG(38:2) | [M+Cl]- | 4.68 |
| 16 | 709.55 | 709.5571 | 709.5537 | 6.17 | DG(40:3) | [M+Cl]- | 4.79 |
| 17 | 711.55 | 711.5728 | 711.5694 | 6.21 | DG(40:2) | [M+Cl]- | 4.77 |
| 18 | 748.55 | 748.5725 | - | 8.41 | Unknown | - | - |
| 19 | 750.55 | 750.588 | 750.5883 | 6.47 | HexCer(d38:3) | [M-H]- | -0.39 |
| 20 | 777.65 | 777.6722 | - | 6.29 | Unknown | - | - |

**Table S4. The 50 ions with the highest intensity in the *m/z* range of 560-1000 were identified using during the ablation of the palatal NKSSE in iKnife-TORS cases.** The ions were putatively identified based on accurate masses using LIPID MAPS with a mass tolerance limit set to +/- 0.01 *m/z.* The underlined bins represent the ions being present in all tissue types *(Cer-Ceramides, CAR-Acyl Carnitines, DG-Diglycerides, PE-Phosphatidylethanolamines, PC- Phosphatidylcholines, HexCer-Hexosyl ceramides).*

| No | Bin | Observed mass | Theoretical mass | Relative abundance x10^3^ | Identity | Adduct | ppm error |
| --- | --- | --- | --- | --- | --- | --- | --- |
| 1 | 572.45 | 572.4808 | 572.4809 | 4.21 | Cer(d34:1) | [M+Cl]- | -0.17 |
| 2 | 573.45 | 573.4839 | 573.4843 | 1.59 | ^13^C isotope of Cer(d34:1) | [M+Cl]- | -0.70 |
| 3 | 574.45 | 574.4902 | 574.4966 | 3.21 | Cer(d34:0) | [M+Cl]- | -11.14 |
| 4 | 575.45 | 575.494 | 575.4999 | 1.04 | ^13^C isotope of Cer(d34:0) | [M+Cl]- | -10.25 |
| 5 | 576.45 | 576.4944 | 576.4954 | 0.75 | 2x ^13^C isotope of Cer(d34:0) | [M+Cl]- | -1.73 |
| 6 | 588.45 | 588.4991 | 588.4997 | 0.46 | CAR(30:3) | [M-H]- | -1.02 |
| 7 | 594.25 | 594.2916 |  | 0.49 | Background noise |  |  |
| 8 | 600.55 | 600.5118 | 600.5122 | 0.72 | Cer(d36:1) | [M+Cl]- | -0.67 |
| 9 | 602.55 | 602.5252 | 602.5279 | 1.17 | Cer(d36:0) | [M+Cl]- | -4.48 |
| 10 | 603.55 | 603.5286 | 603.5312 | 0.42 | ^13^C isotope of Cer(d36:0) | [M+Cl]- | -4.31 |
| 11 | 626.55 | 626.5352 | 626.5279 | 1.07 | Cer(d38:2) | [M+Cl]- | 11.65 |
| 12 | 627.45 | 627.4748 | 627.4761 | 0.74 | DG(34:2) | [M+Cl]- | -2.07 |
| 13 | 627.55 | 627.5383 | 627.5389 | 0.52 | ^13^C isotope of Cer(d38:2) | [M+Cl]- | -0.96 |
| 14 | 628.55 | 628.5477 | 628.5435 | 0.96 | Cer(d38:1) | [M+Cl]- | 6.68 |
| 15 | 629.45 | 629.4893 | 629.4911 | 1.01 | DG(34:1) | [M+Cl]- | -2.86 |
| 16 | 629.55 | 629.5524 | 629.5469 | 0.52 | ^13^C isotope of Cer(d38:1) | [M+Cl]- | 8.74 |
| 17 | 630.55 | 630.5577 | 630.5592 | 0.71 | Cer(d38:0) | [M+Cl]- | -2.38 |
| 18 | 653.45 | 653.4899 | 653.4911 | 0.67 | DG(36:3) | [M+Cl]- | -1.84 |
| 19 | 654.55 | 654.5619 | 654.5592 | 0.44 | Cer(d40:2) | [M+Cl]- | 4.12 |
| 20 | 655.55 | 655.5057 | 655.5068 | 1.71 | DG(36:2) | [M+Cl]- | -1.68 |
| 21 | 656.55 | 656.5092 | 656.5102 | 1.23 | ^13^C isotope of DG(36:2) | [M+Cl]- | -1.52 |
| 22 | 657.55 | 657.5173 | 657.5224 | 2.06 | DG(36:1) | [M+Cl]- | -7.76 |
| 23 | 658.55 | 658.521 | 658.5175 | 1.09 | CerP(d37:1) | [M-H]- | 5.31 |
| 24 | 659.55 | 659.5203 | 659.5209 | 0.59 | ^13^C isotope of CerP(d37:1) | [M+Cl]- | -0.91 |
| 25 | 665.55 | 665.5471 | - | 3.19 | Unknown | - | - |
| 26 | 666.55 | 666.5503 | - | 1.38 | Unknown | - | - |
| 27 | 667.55 | 667.5611 | - | 2.10 | Unknown | - | - |
| 28 | 668.55 | 668.5655 | 668.5748 | 0.87 | Cer(d41:2) | [M+Cl]- | -13.91 |
| 29 | 679.55 | 679.5062 | 679.5068 | 0.68 | DG(38:4) | [M+Cl]- | -0.88 |
| 30 | 681.55 | 681.527 | 681.5224 | 1.24 | DG(38:3) | [M+Cl]- | 6.75 |
| 31 | 682.55 | 682.5863 | 682.5905 | 0.97 | Cer(d42:2) | [M+Cl]- | -6.15 |
| 32 | 683.55 | 683.5432 | 683.5381 | 0.85 | DG(38:2) | [M+Cl]- | 7.46 |
| 33 | 684.55 | 684.5372 | 684.5332 | 0.54 | CerP(d39:2) | [M-H]- | 5.84 |
| 34 | 684.65 | 684.6039 | 684.6061 | 0.58 | Cer(d42:1) | [M+Cl]- | -3.21 |
| 35 | 686.65 | 686.6152 | 686.6218 | 0.61 | Cer(d42:0) | [M+Cl]- | -9.61 |
| 36 | 693.55 | 693.5781 | - | 0.46 | Unknown | - | - |
| 37 | 695.55 | 695.5933 | - | 0.51 | Unknown | - | - |
| 38 | 700.55 | 700.608 | 700.601 | 0.58 | Cer(t42:1) | [M+Cl]- | 9.99 |
| 39 | 709.55 | 709.5617 | 709.5537 | 0.43 | DG(40:3) | [M+Cl]- | 11.27 |
| 40 | 711.55 | 711.5782 | 711.5694 | 0.45 | DG(40:2) | [M+Cl]- | 12.37 |
| 41 | 716.55 | 716.52 | 716.523 | 0.43 | PE(34:1) PC(32:1) | [M-H]- [M-CH3]- | -4.19 |
| 42 | 736.55 | 736.5527 | 736.5494 | 0.42 | HexCer(d34:0) | [M+Cl]- | 4.48 |
| 43 | 742.55 | 742.5384 | 742.5386 | 0.64 | PE(36:2) PC(34:2) | [M-H]- [M-CH3]- | -0.27 |
| 44 | 743.55 | 743.5378 | 743.5381 | 0.42 | DG(43:7) | [M+Cl]- | -0.40 |
| 45 | 744.55 | 744.5502 | 744.5543 | 0.70 | PE(36:1) PC(34:1) | [M-H]- [M-CH3]- | -5.51 |
| 46 | 748.55 | 748.5732 | - | 0.60 | Unknown | - | - |
| 47 | 750.55 | 750.5885 | 750.5883 | 0.85 | HexCer(d38:3) | [M-H]- | 0.27 |
| 48 | 752.55 | 752.5435 | 752.5443 | 0.41 | HexCer(t34:0) | [M+Cl]- | -1.06 |
| 49 | 862.65 | 862.6584 | 862.6538 | 0.56 | HexCer(t42:1) | [M+Cl]- | 5.33 |
| 50 | 864.65 | 864.6674 | 864.6695 | 0.60 | HexCer(t42:0) | [M+Cl]- | -2.43 |

**Table S5. The 50 ions with the highest intensity in the *m/z* range of 560-1000 were identified using during the ablation of the lingual KSSE in iKnife-TORS cases.** The ions were putatively identified based on accurate masses using LIPID MAPS with a mass tolerance limit set to +/- 0.01 *m/z.* The underlined bins represent the ions being present in all tissue types (*Cer-Ceramides, CAR-* *Acyl carnitines, DG-Diglycerides, PA-Phosphatidic acids, LPG - Lyso-Phosphatidylglycerols, PE-Phosphatidylethanolamines, PC- Phosphatidylcholines,* *PG-Phosphatidylglycerols,* *TG-Triglycerides).*

| No | Bin | Observed mass | Theoretical mass | Relative abundance x10^3^ | Identity | Adduct | ppm error |
| --- | --- | --- | --- | --- | --- | --- | --- |
| 1 | 572.45 | 572.4807 | 572.4809 | 0.78 | Cer(d34:1) | [M+Cl]- | 0.35 |
| 2 | 574.45 | 574.483 | 574.4835 | 0.37 | CAR(29:3) | [M-H]- | 0.87 |
| 3 | 594.25 | 594.2866 | - | 0.51 | Background noise | - | - |
| 4 | 612.25 | 612.222 | - | 0.50 | Unknown | - | - |
| 5 | 627.45 | 627.4756 | 627.4755 | 0.46 | DG(34:2) | [M+Cl]- | -0.16 |
| 6 | 629.45 | 629.4899 | 629.4911 | 0.62 | DG(34:1) | [M+Cl]- | 1.91 |
| 7 | 651.45 | 651.4751 | 651.4755 | 0.71 | DG(36:4) | [M+Cl]- | 0.61 |
| 8 | 652.45 | 652.4783 | 652.4789 | 0.33 | ^13^C isotope of DG(36:4) | [M+Cl]- | 0.92 |
| 9 | 653.45 | 653.4841 | 653.4911 | 0.57 | DG(36:3) | [M+Cl]- | 10.71 |
| 10 | 655.55 | 655.5053 | 655.5068 | 0.54 | DG(36:2) | [M+Cl]- | 2.29 |
| 11 | 657.55 | 657.5176 | 657.5224 | 0.62 | DG(36:1) | [M+Cl]- | 7.30 |
| 12 | 661.45 | 661.482 | 661.4808 | 0.32 | PA(33:0) | [M-H]- | -1.81 |
| 13 | 677.45 | 677.4885 | 677.4911 | 0.41 | DG(38:5) | [M+Cl]- | 3.84 |
| 14 | 679.55 | 679.5055 | 679.5068 | 1.13 | DG(38:4) | [M+Cl]- | 1.91 |
| 15 | 680.55 | 680.509 | 680.5102 | 0.66 | ^13^C isotope of DG(38:4) | [M+Cl]- | 1.76 |
| 16 | 681.55 | 681.5111 | - | 0.84 | Unknown | - | - |
| 17 | 682.55 | 682.5888 | 682.5905 | 0.66 | Cer(d42:2) | [M+Cl]- | 2.49 |
| 18 | 683.55 | 683.5926 | 683.5939 | 0.36 | ^13^C isotope of Cer(d42:2) | [M+Cl]- | 1.90 |
| 19 | 691.45 | 691.4914 | 691.4914 | 0.26 | PA(34:0(OH)) | [M-H]- |  |
| 20 | 703.55 | 703.5075 | 703.5068 | 0.41 | DG(40:6) | [M+Cl]- | -1.00 |
| 21 | 705.55 | 705.5149 | 705.507 | 0.38 | LPG(32:1) PA(35:0(OH)) | [M-H]- | -11.20 |
| 22 | 707.55 | 707.5231 | 707.5227 | 0.21 | LPG(32:0) | [M-H]- | -0.57 |
| 23 | 709.45 | 709.48 | 709.4808 | 0.24 | PA(37:4) | [M-H]- | 1.13 |
| 24 | 718.55 | 718.5361 | 718.5386 | 0.50 | PE(34:0) PC(32:0) | [M-H]- [M-CH3]- | 3.48 |
| 25 | 722.55 | 722.5123 | 722.5124 | 0.15 | PE(P-36:4) | [M-H]- | 0.14 |
| 26 | 737.55 | 737.5115 | 737.5121 | 0.26 | PA(39:4) | [M-H]- | 0.81 |
| 27 | 739.45 | 739.494 | 739.4914 | 0.21 | PA(38:4(OH)) | [M-H]- | -3.52 |
| 28 | 742.55 | 742.5389 | 742.5386 | 0.37 | PE(36:2) PC(34:2) | [M-H]- [M-CH3]- | -0.40 |
| 29 | 743.55 | 743.537 | 743.5381 | 0.28 | DG(43:7) | [M+Cl]- | 1.48 |
| 30 | 744.55 | 744.5536 | 744.5543 | 0.50 | PE(36:1) PC(34:1) | [M-H]- [M-CH3]- | 0.94 |
| 31 | 745.55 | 745.5573 | 745.5577 | 0.25 | ^13^C isotope of the PE(36:1) ^13^C isotope of the PC(34:1) | [M-H]-  [M-CH3]- | 0.54 |
| 32 | 746.55 | 746.5123 | 746.5124 | 0.23 | PE(P-38:6) | [M-H]- | 0.13 |
| 33 | 750.55 | 750.5451 | 750.5437 | 0.20 | PE(P-38:4) | [M-H]- | -1.87 |
| 34 | 766.55 | 766.5381 | 766.5386 | 0.63 | PE(38:4) PC(36:4) | [M-H]- [M-CH3]- | 0.65 |
| 35 | 767.55 | 767.5353 | 767.542 | 0.44 | ^13^C isotope of the PE(36:1) ^13^C isotope of the PC(34:1) | [M-H]-  [M-CH3]- | 8.73 |
| 36 | 768.55 | 768.5468 | 768.5451 | 0.29 | ^13^C isotope of the PE(36:1) ^13^C isotope of the PC(34:1) | [M-H]-  [M-CH3]- | -2.21 |
| 37 | 770.55 | 770.5696 | 770.5699 | 0.25 | PE(38:2) PC(36:2) | [M-H]- [M-CH3]- | 0.39 |
| 38 | 792.55 | 792.5533 | 792.5543 | 0.18 | PE(40:5) PC(38:5) | [M-H]- [M-CH3]- | 1.26 |
| 39 | 794.55 | 794.5676 | 794.5699 | 0.26 | PE(40:4) PC(38:4) | [M-H]- [M-CH3]- | 2.89 |
| 40 | 889.75 | 889.7279 | 889.7261 | 0.29 | PG(O-45:0) | [M-H]- | -2.02 |
| 41 | 891.75 | 891.7205 | 891.7208 | 0.69 | TG(52:3) | [M+Cl]- | 0.34 |
| 42 | 892.75 | 892.729 | 892.7289 | 0.37 | CerP(d51:0) | [M+Cl]- | -0.11 |
| 43 | 893.75 | 893.7331 | 893.7364 | 0.68 | TG(52:2) | [M+Cl]- | 3.69 |
| 44 | 894.75 | 894.7355 | 894.7399 | 0.33 | ^13^C isotope of TG(52:2) | [M+Cl]- | 4.92 |
| 45 | 895.75 | 895.7374 | 895.7371 | 0.25 | mixture of isotope of TG(52:2) | [M+Cl]- | -0.33 |
| 46 | 915.75 | 915.7421 | 915.7418 | 0.19 | PG(O-47:1) | [M-H]- | -0.33 |
| 47 | 917.75 | 917.734 | 917.7364 | 0.36 | TG(54:4) | [M+Cl]- | 2.62 |
| 48 | 918.75 | 918.7385 | 918.7399 | 0.19 | ^13^C isotope of TG(54:4) | [M+Cl]- | 1.52 |
| 49 | 919.75 | 919.7509 | 919.7521 | 0.35 | TG(54:3) | [M+Cl]- | 1.30 |
| 50 | 920.75 | 920.7537 | 920.7555 | 0.19 | ^13^C isotope of TG(54:3) | [M+Cl]- | 1.95 |

**Table S6. The 50 ions with the highest intensity in the *m/z* range of 560-1000 were identified using during the ablation of the epiglottic NKSSE in iKnife-TORS cases.** The ions were putatively identified based on accurate masses using LIPID MAPS with a mass tolerance limit set to +/- 0.01 *m/z.* The underlined bins represent the ions being present in all tissue types (*Cer-Ceramides, CAR- Acyl carnitines, DG-Diglycerides, PE-Cer- Phosphatidylethanolamine-Ceramides, SM-Sphingomyelins, PE-Phosphatidylethanolamines, PC- Phosphatidylcholines, TG-Triglycerides).*

| No | Bin | Observed mass | Theoretical mass | Relative abundance x10^3^ | Identity | Adduct | ppm error |
| --- | --- | --- | --- | --- | --- | --- | --- |
| 1 | 572.45 | 572.4804 | 572.4809 | 1.24 | Cer(d34:1) | [M+Cl]- | 0.87 |
| 2 | 573.45 | 573.4844 | 573.4843 | 0.49 | ^13^C isotope of Cer(d34:1) | [M+Cl]- | -0.17 |
| 3 | 574.45 | 574.4848 | 574.4835 | 0.67 | CAR(29:3) | [M-H]- | -2.26 |
| 4 | 576.25 | 576.2433 | - | 1.03 | Background noise | - | - |
| 5 | 594.25 | 594.4067 | - | 1.21 | Background noise | - | - |
| 6 | 595.25 | 595.4108 | - | 0.55 | Background noise | - | - |
| 7 | 611.25 | 611.278 | - | 0.54 | Background noise | - | - |
| 8 | 612.25 | 612.2207 | - | 1.15 | Unknown | - | - |
| 9 | 627.45 | 627.4754 | 627.4755 | 0.63 | DG(34:2) | [M+Cl]- | 0.16 |
| 10 | 629.45 | 629.489 | 629.4911 | 0.71 | DG(34:1) | [M+Cl]- | 3.34 |
| 11 | 651.45 | 651.4739 | 651.4755 | 0.48 | DG(36:4) | [M+Cl]- | 2.46 |
| 12 | 653.45 | 653.488 | 653.4911 | 0.52 | DG(36:3) | [M+Cl]- | 4.74 |
| 13 | 655.55 | 655.5062 | 655.5068 | 0.69 | DG(36:2) | [M+Cl]- | 0.92 |
| 14 | 656.55 | 656.5098 | 656.5102 | 0.50 | ^13^C isotope of DG(36:2) | [M+Cl]- | 0.61 |
| 15 | 657.55 | 657.5177 | 657.5224 | 0.94 | DG(36:1) | [M+Cl]- | 7.15 |
| 16 | 658.55 | 658.5217 | 658.5175 | 0.40 | CerP(d37:1) | [M+Cl]- | -6.38 |
| 17 | 679.55 | 679.5057 | 679.5068 | 0.62 | DG(38:4) | [M+Cl]- | 1.62 |
| 18 | 680.55 | 680.5093 | 680.5102 | 0.41 | ^13^C isotope of DG(38:4) | [M+Cl]- | 1.32 |
| 19 | 681.55 | 681.5137 | 681.5224 | 0.54 | DG(38:3) | [M+Cl]- | 12.77 |
| 20 | 682.55 | 682.5891 | 682.5905 | 0.65 | Cer(d42:2) | [M+Cl]- | 2.05 |
| 21 | 687.55 | 687.5439 | 687.5441 | 0.39 | PE-Cer(d36:1) SM(d34:1) | [M-H]- [M-CH3]- | 0.29 |
| 22 | 718.55 | 718.5354 | 718.5386 | 0.37 | PE(34:0) PC(32:0) | [M-H]- [M-CH3]- | 4.45 |
| 23 | 742.55 | 742.5388 | 742.5386 | 0.83 | PE(36:2) PC(34:2) | [M-H]- [M-CH3]- | -0.27 |
| 24 | 743.55 | 743.5371 | 743.5381 | 0.58 | DG(43:7) | [M+Cl]- | 1.34 |
| 25 | 744.55 | 744.5536 | 744.5543 | 0.80 | PE(36:1) PC(34:1) | [M-H]- [M-CH3]- | 0.94 |
| 26 | 745.55 | 745.5558 | 745.5577 | 0.38 | ^13^C isotope of the PE(36:1) ^13^C isotope of the PC(34:1) | [M-H]-  [M-CH3]- | 2.55 |
| 27 | 750.55 | 750.5444 | 750.5437 | 0.38 | PE(P-38:4) | [M-H]- | -0.93 |
| 28 | 766.55 | 766.5388 | 766.5386 | 0.49 | PE(38:4) PC(36:4) | [M-H]- [M-CH3]- | -0.26 |
| 29 | 768.55 | 768.5515 | 768.5543 | 0.33 | PE(38:3) PC(36:3) | [M-H]- [M-CH3]- | 3.64 |
| 30 | 770.55 | 770.57 | 770.5699 | 0.45 | PE(38:2) PC(36:2) | [M-H]- [M-CH3]- | -0.13 |
| 31 | 865.75 | 865.7026 | 865.7051 | 0.62 | TG(50:2) | [M+Cl]- | 2.89 |
| 32 | 867.75 | 867.7133 | 867.7208 | 0.52 | TG(50:1) | [M+Cl]- | 8.64 |
| 33 | 889.75 | 889.7069 | 889.7051 | 1.28 | TG(52:4) | [M+Cl]- | -2.02 |
| 34 | 890.75 | 890.706 | 890.7086 | 0.93 | ^13^C isotope of TG(52:4) | [M+Cl]- | 2.92 |
| 35 | 891.75 | 891.7203 | 891.7208 | 3.45 | TG(52:3) | [M+Cl]- | 0.56 |
| 36 | 892.75 | 892.723 | 892.7242 | 1.97 | ^13^C isotope of TG(52:3) | [M+Cl]- | 1.34 |
| 37 | 893.75 | 893.7318 | 893.7364 | 3.60 | TG(52:2) | [M+Cl]- | 5.15 |
| 38 | 894.75 | 894.7355 | 894.7399 | 1.90 | ^13^C isotope of TG(52:2) | [M+Cl]- | 4.92 |
| 39 | 895.75 | 895.7374 | 895.7371 | 1.36 | mixture of isotope TG(52:2) | [M+Cl]- | -0.33 |
| 40 | 896.75 | 896.7397 | 896.7386 | 0.51 | mixture of isotope TG(52:2) | [M+Cl]- | -1.23 |
| 41 | 915.75 | 915.7197 | 915.7208 | 1.00 | TG(54:5) | [M+Cl]- | 1.20 |
| 42 | 916.75 | 916.7234 | 916.7242 | 0.64 | ^13^C isotope of TG(54:5) | [M+Cl]- | 0.87 |
| 43 | 917.75 | 917.7352 | 917.7364 | 1.82 | TG(54:4) | [M+Cl]- | 1.31 |
| 44 | 918.75 | 918.7388 | 918.7399 | 1.00 | ^13^C isotope of TG(54:4) | [M+Cl]- | 1.20 |
| 45 | 919.75 | 919.748 | 919.7521 | 1.78 | TG(54:3) | [M+Cl]- | 4.46 |
| 46 | 920.75 | 920.7514 | 920.7555 | 0.95 | ^13^C isotope of TG(54:3) | [M+Cl]- | 4.45 |
| 47 | 921.75 | 921.7559 | 921.7529 | 0.81 | mixture of isotopes TG(54:3) | [M+Cl]- | -3.25 |
| 48 | 939.75 | 939.7196 | 939.7208 | 0.37 | TG(56:7) | [M+Cl]- | 1.28 |
| 49 | 941.75 | 941.7323 | 941.7364 | 0.49 | TG(56:6) | [M+Cl]- | 4.35 |
| 50 | 943.75 | 943.7455 | 943.7521 | 0.36 | TG(56:5) | [M+Cl]- | 6.99 |

**Table S7. The 40 ions with the highest intensity in the *m/z* range of 600-1000 identified using during the iKnife-parathyroidectomy case.** The ions were putatively identified based on accurate masses using LIPID MAPS with a mass tolerance limit set to +/- 0.01 *m/z.* ( *DG-Diglycerides, SM-Sphingomyelins, TG-Triglycerides).*

| No | Bin | Observed mass | Theoretical mass | Relative abundance x10^3^ | Identity | Adduct | ppm error |
| --- | --- | --- | --- | --- | --- | --- | --- |
| 1 | 611.25 | 611.2832 | - | 1.21 | Background noise | - | - |
| 2 | 612.25 | 612.22 | - | 2.20 | Unknown | - | - |
| 3 | 613.25 | 613.2227 | - | 0.70 | Background noise | - | - |
| 4 | 614.25 | 614.2172 | - | 0.74 | Background noise | - | - |
| 5 | 627.45 | 627.4752 | 627.4755 | 0.77 | DG(34:2) | [M+Cl]- | 0.48 |
| 6 | 629.45 | 629.4886 | 629.4911 | 1.21 | DG(34:1) | [M+Cl]- | 3.97 |
| 7 | 630.45 | 630.492 | 630.4945 | 0.42 | ^13^C isotope DG(34:1) | [M+Cl]- | 3.97 |
| 8 | 631.45 | 631.4904 | 631.4903 | 0.46 | 2x ^13^C isotope of DG(34:1) | [M+Cl]- | -0.16 |
| 9 | 639.25 | 639.2389 | - | 0.83 | Background noise | - | - |
| 10 | 653.45 | 653.4891 | 653.4865 | 0.47 | DG(36:3) | [M+Cl]- | -3.98 |
| 11 | 655.55 | 655.504 | 655.5049 | 0.63 | DG(36:2) | [M+Cl]- | 1.37 |
| 12 | 657.55 | 657.5181 | 657.5181 | 0.85 | DG(36:1) | [M+Cl]- | 0.00 |
| 13 | 666.25 | 666.2755 | - | 1.23 | Background noise | - | - |
| 14 | 837.65 | 837.6724 | 837.6738 | 0.52 | TG(48:2) | [M+Cl]- | 1.67 |
| 15 | 839.65 | 839.6859 | 839.6895 | 0.66 | TG(48:1) | [M+Cl]- | 4.29 |
| 16 | 863.65 | 863.6886 | 863.6895 | 0.52 | TG(50:3) | [M+Cl]- | 1.04 |
| 17 | 865.65 | 865.6886 | 865.6929 | 0.73 | SM(d43:0) | [M+Cl]- | 4.97 |
| 18 | 865.75 | 865.7037 | 865.7051 | 1.03 | TG(50:2) | [M+Cl]- | 1.62 |
| 19 | 866.75 | 866.7076 | 866.7086 | 0.65 | ^13^C isotope of TG(50:2) | [M+Cl]- | 1.15 |
| 20 | 867.75 | 867.7163 | 867.7208 | 1.63 | TG(50:1) | [M+Cl]- | 5.19 |
| 21 | 868.75 | 868.7202 | 868.7242 | 0.90 | ^13^C isotope of TG(50:1) | [M+Cl]- | 4.60 |
| 22 | 869.75 | 869.7219 | 869.7213 | 0.67 | mixture of isotope TG(50:1) | [M+Cl]- | -0.69 |
| 23 | 889.75 | 889.7049 | 889.7051 | 0.93 | TG(52:4) | [M+Cl]- | 0.22 |
| 24 | 890.75 | 890.7087 | 890.7086 | 0.61 | ^13^C isotope of TG(52:4) | [M+Cl]- | -0.11 |
| 25 | 891.75 | 891.7197 | 891.7203 | 4.17 | TG(52:3) | [M+Cl]- | 0.67 |
| 26 | 892.75 | 892.7238 | 892.723 | 2.53 | ^13^C isotope of TG(52:3) | [M+Cl]- | -0.90 |
| 27 | 893.75 | 893.7325 | 893.7318 | 5.85 | TG(52:2) | [M+Cl]- | -0.78 |
| 28 | 894.75 | 894.7368 | 894.7355 | 3.27 | ^13^C isotope of TG(52:2) | [M+Cl]- | -1.45 |
| 29 | 895.75 | 895.7398 | 895.7374 | 2.75 | mixture of isotope TG(52:2) | [M+Cl]- | -2.68 |
| 30 | 896.75 | 896.7424 | 896.7397 | 1.21 | mixture of isotope TG(52:2) | [M+Cl]- | -3.01 |
| 31 | 897.75 | 897.7491 | 897.7411 | 0.49 | mixture of isotope TG(52:2) | [M+Cl]- | -8.91 |
| 32 | 915.75 | 915.7203 | 915.7197 | 0.91 | TG(54:5) | [M+Cl]- | -0.66 |
| 33 | 916.75 | 916.7239 | 916.7234 | 0.53 | ^13^C isotope of TG(54:5) | [M+Cl]- | -0.55 |
| 34 | 917.75 | 917.7351 | 917.7352 | 2.64 | TG(54:4) | [M+Cl]- | 0.11 |
| 35 | 918.75 | 918.7389 | 918.7388 | 1.56 | ^13^C isotope of TG(54:4) | [M+Cl]- | -0.11 |
| 36 | 919.75 | 919.7487 | 919.748 | 3.66 | TG(54:3) | [M+Cl]- | -0.76 |
| 37 | 920.75 | 920.7531 | 920.7514 | 2.08 | ^13^C isotope of TG(54:3) | [M+Cl]- | -1.85 |
| 38 | 921.75 | 921.7587 | 921.7559 | 2.27 | mixture of isotopes TG(54:3) | [M+Cl]- | -3.04 |
| 39 | 922.75 | 922.7628 | 922.7622 | 1.12 | mixture of isotopes TG(54:3) | [M+Cl]- | -0.65 |
| 40 | 923.75 | 923.7678 | 923.7656 | 0.73 | mixture of isotopes TG(54:3) | [M+Cl]- | -2.38 |

**Table S8. Details of the surgical robotic cases acquired using the iKnife instrumentation**

|  | DIAGNOSIS | PROCEDURE | GENDER | AGE |
| --- | --- | --- | --- | --- |
| 1 | Obstructive Sleep apnea | Uvulopalatopharyngoplasty  TORS to tongue base and soft palate | Male | 51 |
| 2 | Obstructive Sleep apnea | Uvulopalatopharyngoplasty  TORS to palate, tongue base, and epiglottis | Male | 52 |
| 3 | Hyperparathyroidism – Parathyroid adenoma | Robotic Transaxillary Left Superior Parathyroidectomy | Female | 46 |

**Table S9. Details on how each tissue type affect various spectral characteristics such as the TIC, signal intensities, background noise and signal-to-noise ratios in the *m/z* range of 560-1000. ‘Signal’ is defined as the total intensity of the 20 highest peaks in the specific *m/z* range and ‘Noise’ is defined as the median intensity of peaks within the same *m/z* range.**

| ***m/z* range** | **Variables** | **Spectra** | **Mean TIC x10^7^ (range)** | **Mean Signal x10^-1^  (range)** | **Mean Noise x10^-5^ (range)** | **Mean SNR x10^3^ (range)** |
| --- | --- | --- | --- | --- | --- | --- |
|  | palate | 64 | 1.14 (0.41-2.16) | 0.87 (0.41-2.37) | 7.67 (4.87-11.2) | 1.23 (0.43-4.42) |
| **560-1000** | tongue | 28 | 0.87 (0.40-1.58) | 0.58 (0.36-1.0) | 9.73 (4.36-13.7) | 0.73 (0.30-2.1) |
|  | epiglottis | 13 | 0.94 (0.23-1.68) | 1.15 (0.86-1.83) | 8.01 (5.3-14.1) | 4.75 (0.70-4.01) |

**Supplementary movie legends**

**Movie S1.** Soft palate ablation using a 2 µm thulium laser and extraction of the generated surgical aerosol using the suction tool during the iKnife-TORS cases.

**Movie S2.** Dissection of the parathyroid adenoma using the Harmonic scalpel during the Robotic Transaxillary iKnife-Parathyroidectomy case.

**References**

1. Schäfer, K.-C.; Balog, J.; Szaniszló, T.; Szalay, D.; Mezey, G.; Dénes, J.; Bognár, L.; Oertel, M.; Takáts, Z., Real Time Analysis of Brain Tissue by Direct Combination of Ultrasonic Surgical Aspiration and Sonic Spray Mass Spectrometry. *Analytical Chemistry* **2011,** *83* (20), 7729-7735.

2. Balog, J.; Kumar, S.; Alexander, J.; Golf, O.; Huang, J.; Wiggins, T.; Abbassi-Ghadi, N.; Enyedi, A.; Kacska, S.; Kinross, J.; Hanna, G. B.; Nicholson, J. K.; Takats, Z., In Vivo Endoscopic Tissue Identification by Rapid Evaporative Ionization Mass Spectrometry (REIMS). *Angewandte Chemie International Edition* **2015,** *54* (38), 11059-11062.

3. Tzafetas, M.; Mitra, A.; Paraskevaidi, M.; Bodai, Z.; Kalliala, I.; Bowden, S.; Lathouras, K.; Rosini, F.; Szasz, M.; Savage, A.; Balog, J.; McKenzie, J.; Lyons, D.; Bennett, P.; MacIntyre, D.; Ghaem-Maghami, S.; Takats, Z.; Kyrgiou, M., The intelligent knife (iKnife) and its intraoperative diagnostic advantage for the treatment of cervical disease. *Proceedings of the National Academy of Sciences* **2020,** *117* (13), 7338.

4. Vogel, A.; Venugopalan, V., Mechanisms of pulsed laser ablation of biological tissues. *Chem Rev* **2003,** *103* (2), 577-644.

5. Schäfer, K.-C.; Szaniszló, T.; Günther, S.; Balog, J.; Dénes, J.; Keserű, M.; Dezső, B.; Tóth, M.; Spengler, B.; Takáts, Z., In Situ, Real-Time Identification of Biological Tissues by Ultraviolet and Infrared Laser Desorption Ionization Mass Spectrometry. *Analytical Chemistry* **2011,** *83* (5), 1632-1640.

6. Wijnant, K.; Van Meulebroek, L.; Pomian, B.; De Windt, K.; De Henauw, S.; Michels, N.; Vanhaecke, L., Validated Ultra-High-Performance Liquid Chromatography Hybrid High-Resolution Mass Spectrometry and Laser-Assisted Rapid Evaporative Ionization Mass Spectrometry for Salivary Metabolomics. *Analytical Chemistry* **2020,** *92* (7), 5116-5124.

7. Temelkuran, B.; Hart, S. D.; Benoit, G.; Joannopoulos, J. D.; Fink, Y., Wavelength-scalable hollow optical fibres with large photonic bandgaps for CO2 laser transmission. *Nature* **2002,** *420* (6916), 650-653.

8. Harrington, J. A., A Review of IR Transmitting, Hollow Waveguides. *Fiber and Integrated Optics* **2000,** *19* (3), 211-227.

9. Karaman, M.; Gün, T.; Temelkuran, B.; Aynacı, E.; Kaya, C.; Tekin, A. M., Comparison of fiber delivered CO(2) laser and electrocautery in transoral robot assisted tongue base surgery. *Eur Arch Otorhinolaryngol* **2017,** *274* (5), 2273-2279.

10. Warnaby, C. E.; Coleman, D. J.; King, T. A. In *Photothermal modelling of thulium fibre laser-tissue interactions*, Therapeutic Laser Applications and Laser-Tissue Interactions, Munich, 2003/06/22; Steiner, R., Ed. Optical Society of America: Munich, 2003; p 5142_68.

11. Zeitels, S. M.; Burns, J. A.; Akst, L. M.; Hillman, R. E.; Broadhurst, M. S.; Anderson, R. R., Office-based and microlaryngeal applications of a fiber-based thulium laser. *Ann Otol Rhinol Laryngol* **2006,** *115* (12), 891-6.

12. Wiklund, S.; Johansson, E.; Sjöström, L.; Mellerowicz, E. J.; Edlund, U.; Shockcor, J. P.; Gottfries, J.; Moritz, T.; Trygg, J., Visualization of GC/TOF-MS-Based Metabolomics Data for Identification of Biochemically Interesting Compounds Using OPLS Class Models. *Analytical Chemistry* **2008,** *80* (1), 115-122.
